# Raftophilic rhodopsin-clusters offer stochastic platforms for G protein signalling in retinal discs

**DOI:** 10.1038/s42003-019-0459-6

**Published:** 2019-06-14

**Authors:** Fumio Hayashi, Natsumi Saito, Yasushi Tanimoto, Keisuke Okada, Kenichi Morigaki, Keiji Seno, Shohei Maekawa

**Affiliations:** 1 Graduate School of Science, Kobe University, 1-1 Rokkodai, Nada, Kobe, 657-8501 Japan; 2 Research Centre for Environmental Genomics, Kobe University, 1-1 Rokkodai, Nada, Kobe, 657-8501 Japan; 3 Graduate School of Agriculture, Kobe University, 1-1 Rokkodai, Nada, Kobe, 657-8501 Japan; 4 Faculty of Medicine, Hamamatsu University School of Medicine, 1-20-1 Handayama, Higashi-ku, Hamamatsu, Shizuoka, 431-3192 Japan; 5 International Mass Imaging Centre, Hamamatsu University School of Medicine, 1-20-1 Handayama, Higashi-ku, Hamamatsu, Shizuoka, 431-3192 Japan

**Keywords:** Membrane structure and assembly, Sensory processing, Computational biophysics

## Abstract

Rhodopsin is a G protein-coupled receptor (GPCR) that initiates the phototransduction cascade in retinal disc membrane. Recent studies have suggested that rhodopsin forms highly ordered rows of dimers responsible for single-photon detection by rod photoreceptors. Dimerization is also known to confer to rhodopsin a high affinity for ordered lipids (raftophilicity). However, the role of rhodopsin organization and its raftophilicity in phototransduction remains obscure, owing to the lack of direct observation of rhodopsin dynamics and distribution in native discs. Here, we explore the single-molecule and semi-multimolecule behaviour of rhodopsin in native discs. Rhodopsin forms transient meso-scale clusters, even in darkness, which are loosely confined to the disc centre. Cognate G protein transducin co-distributes with rhodopsin, and exhibits lateral translocation to the disc periphery upon activation. We demonstrate that rhodopsin offers inherently distributed and stochastic platforms for G protein signalling by self-organizing raftophilic clusters, which continually repeat generation/extinction in the disc membrane.

## Abstract (web summary)

Fumio Hayashi et al. show that rhodopsin forms transient clusters in retinal disk membranes using single molecule tracking with near-infrared wavelength. They find that the raftophobic disc periphery excludes rhodopsin clusters and transient rhodopsin-clusters centrally confined in the membrane provide stochastic platforms for G protein signaling.

## Introduction

G-protein-coupled receptors (GPCRs) represent the third largest family of genes in the human genome. Extensive studies have been carried out on the structure and function of GPCRs, and now have been extended to investigations into the functional significance of their dimerization or higher oligomerization^1–3^. Oligomerization of GPCRs has the potential to affect all aspects of the signalling cycle, including receptor biogenesis, activation and desensitization^1^. Furthermore, attention has been drawn to the membrane-mediated oligomerization and related compartmentalization of GPCRs into membrane nanodomains, i.e. rafts or caveolae, and to the implications of such nanodomains for GPCR functions^2^. The nanodomains are characterized by their “raftophilicity”^4^, i.e., a favourability to ordered lipids in the liquid-ordered (*L*_o_) state^5^, and segregated from the more loosely organized bulk lipid bilayer in the liquid-disordered (*L*_d_) state.

Vertebrate phototransduction machinery is a prototypical G-protein-signalling system, extensively studied and thought to be fully understood. In the classical scenario, the signalling processes are explained by the diffusion-collision coupling between key players, i.e. the photopigment rhodopsin, cognate trimeric G protein transducin (G_t_) and its target enzyme 3′,5′-cGMP-phosphodiesterase (PDE6)^6^. Photoisomerized rhodopsin (Rh*) binds to G_t_ to form a 2:1 complex (Rh*_2_–G_t_ complex)^7,8^. In the presence of guanosine-5′-triphosphate (GTP), Rh* catalytically activates ~10 G_t_ molecules within the lifetime of Rh*^9,10^. Following nucleotide-exchange on the α-subunit of G_t_ (Gα_t_), activated Gα_t_ starts to diffuse across the membrane to activate its target enzyme, PDE6. The rhodopsin/G_t_/PDE6 ratio in the disc membrane is set at approximately 100:10: > 1^11^. The cGMP-hydrolysis results in hyperpolarization of the plasma membrane by closing cyclic nucleotide-gated ion channels. These processes have been explained by the diffusion-based coupling of membrane proteins^11^. However, the theory regarding the physical background upon which these processes occur has recently been challenged. Atomic force microscopy (AFM) revealed a para-crystalline arrangement of rhodopsin dimers^12^, suggesting a lower degree of lateral diffusion of rhodopsin in the disc. Subsequent AFM studies have shown meso-sized “nanodomains” without internal structure, which are loosely confined in the central area of the disc membrane^13–15^. A cryo-electron tomographic study has shown that at least ten rhodopsin dimers form pairs of rows (tracks) aligned parallel to the disc incisures^10^, and their accompanying simulation results suggested that the track structure can explain the uniform single-photon response in rod photoreceptors, a long-standing question in phototransduction studies^16^. Although such structural studies have provided static pictures of the supramolecular structure of rhodopsin, a coarse-grained molecular-dynamics simulation study implied that the rhodopsin organization would be formed through relatively weak (1.2–3.6 kcal/mol) protein−protein interactions, via multiple dimerization interfaces, and that the organization should be transient^17^. In addition, accumulated evidence indicating that the average diffusion coefficient of rhodopsin is in the range of 0.1–0.6 µm^2^ s^−1^ ^18–24^ strongly suggests that rhodopsin molecules are diffusive in the discs. Moreover, we should note that rhodopsin is expected to be in dynamic equilibrium between monomers, dimers, and higher-order oligomers^25–27^ and that the transition between these states is regulated by the balance of protein−lipid, protein−protein, and lipid−lipid interactions^25^.

The disc membrane is also known to have another type of inhomogeneity, presumably based on the raftophilicity of oligomerized-rhodopsin. We and others have already found that 10–30% of rhodopsin in the dark-adapted disc membrane is recovered in the detergent-resistant membrane (DRM)^28,29^, the distribution of which is quite a useful index of raftophilicity for membrane proteins^30^. Furthermore, G_t_ exhibits noticeable translocation to DRM from detergent-soluble membrane when rhodopsin is isomerized^28^, suggesting that Rh* activates G_t_ in the raftophilic membrane domain in the disc. These results suggest that there are rhodopsin-containing raftophilic membrane domains in the disc membrane, where Rh* activates G_t_. Our subsequent study revealed that the G_t_-stabilized dimer of rhodopsin is responsible for the raftophilicity of the Rh*–G_t_ complex, and that palmitoyl modification of rhodopsin is a prerequisite for raftophilicity attained by dimerization^8^.

To explore such raftophilicity-based membrane inhomogeneity, previous studies on the raft of the plasma membrane provide important clues^4,30,31^. The ‘raft’ has been defined as a molecular complex consisting of at least three molecules, including a molecule with a saturated alky chain or a cholesterol molecule that plays a critical role in the creation of the complex itself^4^. The raft in the plasma membrane of an unstimulated cell is generally short-lived and nano-sized^4^. However, when transmembrane proteins having raftophilic moiety (e.g., glycosylphosphatidylinositol-anchored receptor protein (GPI-AR)) are oligomerized with ligand or immunoglobulin G (IgG), they form meso-sized, long-lived raftophilic receptor-clusters, the so-called ‘receptor-cluster rafts’^4^, in which raftophilic lipids and proteins are recruited to stabilize the cluster raft. Here we should note that rhodopsin, like GPI-AR, has two saturated alkyl chains (di-palmitoyl)^32^, which provides a strong raft-targeting mechanism for transmembrane proteins^33^. Despite palmitoylation, monomeric rhodopsin is non-raftophilic (raftophobic) and excluded from the *L*_o_-phase in a cholesterol-dependent manner^34^. On the contrary, like GPI-AR, rhodopsin attains a high raftophilicity upon stabilized-dimerization^8^. Therefore, the very close resemblance between GPI-AR and rhodopsin leads us to hypothesize that rhodopsin forms a receptor-cluster raft when G_t_ or IgG stabilizes a rhodopsin dimer. Furthermore, the presence of a considerable population of rhodopsin in the DRM of dark-adapted discs also implies that rhodopsin spontaneously forms receptor-cluster rafts even in darkness.

Here we aim to confirm the presence of dynamic inhomogeneity due to transient formation of rhodopsin-cluster rafts in native disc membrane. We explore the single-molecule behaviour of rhodopsin and transducin in native disc membrane by single-molecule tracking with a near-infrared (near-IR) wavelength. Applying variational Bayes hidden Markov model (HMM) analysis^35,36^ to the single-molecule diffusion of rhodopsin, we derive the number of diffusion states of rhodopsin and the transition rates between them, and examine the effect of light, GTP and cholesterol-depletion on them. In addition, we explore the collective behaviour of rhodopsin and other membrane molecules using the “semi-multimolecule fluorescence imaging” technique that we have developed. Our results clearly show that rhodopsin forms transient meso-sized raftophilic clusters, loosely confined in the disc membrane, which are excluded from the raftophobic disc periphery. The stochastic nature of the physical background for phototransduction is revealed.

## Results

### Single-molecule tracking study of rhodopsin in frog native discs

We first investigated whether rhodopsin forms dynamic clusters in dark-adapted native disc membranes of bullfrog (*Rana catesbeiana*), by performing single-molecule tracking of rhodopsin and transducin on a total internal fluorescence microscope (TIRFM) using a near-IR wavelength. Assuming that dynamic clustering transiently retards the diffusion of rhodopsin, we subjected the single-molecule tracking data to variational Bayes hidden Markov model (HMM) analysis^35^, to infer the number of diffusive states and transition rates between them^36^.

**Fig. 1:**
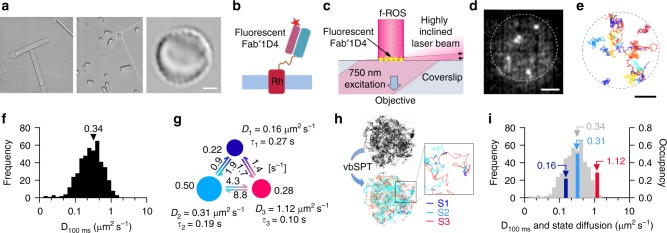
Single-molecule tracking of rhodopsin in rod outer segment (ROS) disc membranes. **a** Left: Intact ROSs associated with rod inner segments. Middle: mechanically fragmented ROSs (f-ROSs). Right: Disc membrane exposed at the bottom of an f-ROS. **b** Schematic diagram for probing rhodopsin (Rh) in the disc membrane. **c** Visualization of single fluorescent spots on the disc membrane, at the bottom of an f-ROS, standing on a glass surface placed on a total internal reflection fluorescence microscope (TIRFM) equipped with electron-multiplying-charge coupled device (EM-CCD) camera. **d** Snapshot of a disc incubated with 0.2 nM fluorescent Fab′-1D4. **e** Representative trajectories of rhodopsin on a dark-adapted disc under physiological conditions. Individual trajectories are colour-coded. Scale bars for both (**d**) and (**e**): 2 μm. **f** Histogram of *D*_100 ms_ calculated from 432 trajectories of rhodopsin. **g** Optimal three-state HMM of rhodopsin inferred by vbSPT. Diffusion coefficients (*D*_1−3_), dwell times (*τ*_1−3_), and relative occupancies of three states (S1, S2 and S3) and the transition rates between them are indicated. Circle sizes reflect relative occupancy. **h** A subset of 100 trajectories is colour-coded according to diffusive state. Square-region is enlarged. **i** Diffusion coefficients (*D*_state_) and occupancies of three diffusive states in an optimal HMM of dark-state rhodopsin are indicated in *D*_100 ms_-histogram (grey bars) (navy bar: state-1, blue bar: state-2, magenta bar: state-3). Coloured numbers are *D*_state_. Grey arrowhead indicates the median value of *D*_100 ms_

As the specimen, we used disc membranes (~8 µm in diameter) exposed at the end of mechanically fragmented rod outer segments (f-ROSs) from frog rod photoreceptors (Fig. 1a). Rhodopsin was probed with the Fab′ fragment of the anti-rhodopsin monoclonal antibody 1D4 (Fab′–1D4) (Supplementary Fig. 1a) labelled with a near-IR dye (Fig. 1b). Fluorescently labelled Fab′–1D4 on the disc membrane was illuminated with a highly inclined laser beam (750-nm in wavelength), almost parallel to the glass surface on a TIRFM (Fig. 1c), and single-molecule fluorescent spots were observed (Fig. 1d). This approach matches that called variable-angle evanescent microscopy^37^ or ‘pseudo-TIRF’^38^ (Fig. 1c and Supplementary Fig. 2a).

Our first finding was that all fluorescently labelled rhodopsin molecules were mobile (Fig. 1e and Supplementary Movie 1). If the disc membrane contacts the glass surface, lateral diffusion of membrane proteins should be hindered. However, we found that there is a gap of about 280 nm between the bottom of the f-ROS and the glass surface (Supplementary Fig. 2a). The gap is likely filled with cytoplasm leached from the cut end of the f-ROS. Furthermore, it is highly likely that what we saw were fluorescent probes bound to the most-accessible disc surface, exposed at the bottom of f-ROS, since we did not see fluorescent spots when we moved the focus up above ~40 nm (almost one increment on fine-focus dial, corresponding to 1–2 disc stacks) from the bottom-disc surface (Supplementary Fig. 2a). In addition, the intensity distribution of fluorescent spots was almost a single Gaussian (Supplementary Fig. 2b), eliminating the possibility of bright spot agglutination due to the contact of disc with the glass surface.

To determine the effective microscopic lateral diffusion coefficient of rhodopsin in the disc, we used mean square displacement (MSD)-time interval plot analysis^39^. The effective microscopic diffusion coefficients of rhodopsin in an interval of 100 ms (*D*_100 ms_) produced a broad single-peak histogram (Fig. 1f). The median value was in good agreement with previously measured macroscopic rhodopsin diffusion coefficients in discs^18–24^. The open-source software for variational Bayes single-particle tracking, i.e. vbSPT^36^ indicated a three-state optimal HMM for rhodopsin diffusion in dark-adapted discs (Fig. 1g; the complete results of the vbSPT analyses are given in Supplementary Fig. 3; the statistical credibility of the inference on the three-state HMM of Fig. 1g is shown in Supplementary Table 1). Trajectories can be colour-coded on the basis of diffusive state (Fig. 1h). The three diffusive states of rhodopsin are shown in the histogram of *D*_100 ms_ (Fig. 1i). There seemingly was a contradiction between the high occupancy of fast diffusive state-3 and the modest distribution of *D*_100 ms_ in the corresponding highly diffusive range. That contradiction is due to the conceptual difference between the diffusion coefficients calculated by MSD analysis and by vbSPT analysis. Whereas MSD analysis relies on time-ensemble averaging, vbSPT analysis relies on time-series analysis assuming memory-less jumps between diffusive states. The former gives the time-averaged diffusion coefficient of a molecule that undergoes diffusion by transitioning through multiple diffusive states, whereas the latter gives the inherent diffusion coefficient of each diffusive state.

### Effect of light and GTP on rhodopsin diffusivity

**Fig. 2:**
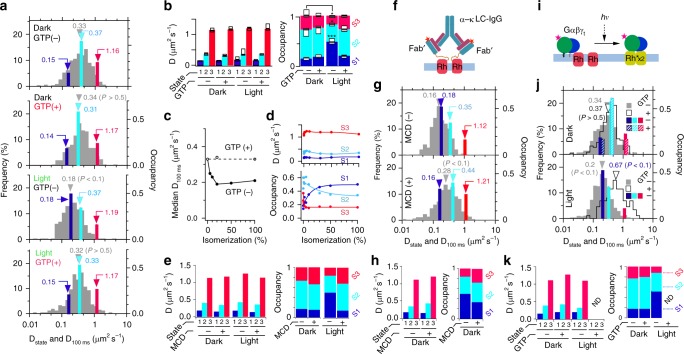
vbSPT analysis of the diffusion of rhodopsin and Gα_t_ in disc membrane. **a** Effect of light and GTP on the optimal HMM of rhodopsin diffusion. Coloured bars, shown in *D*_100 ms_-histograms (grey bars), indicate *D*_state_ and occupancies in a representative optimal three-state HMM of rhodopsin calculated from single-molecule tracking data of ~400 trajectories with ~35-frames length on average. Top and upper-middle panels are from dark-adapted discs without or with GTP, respectively. Lower-middle and bottom panels are from light-exposed discs (20% of rhodopsin isomerized) without or with GTP, respectively. The HMM data are means of three experiments. Grey arrowheads indicate medians of *D*_100 ms_. Significant differences (*P*) within histograms, vs. the top panel, by Mann−Whitney *U* test are indicated. **b** Bar graph of *D*_state_ (left) and stacked bar graph of state occupancies (right) indicating mean ± SD (*N* = 3). Statistically significant differences by two-way ANOVA are labelled. *P < 0.05, ***P < 0.001 vs. corresponding state in the dark in the absence of GTP, by Tukey’s multiple-comparison test. **c** Light-dose-dependence of rhodopsin’s *D*_100 ms_ in the presence or absence of GTP. **d** Light-dose-dependence of rhodopsin’s *D*_state_ (upper) and occupancies (lower). **e** Effect of 20 mM MCD on *D*_state_ and occupancies in HMM of rhodopsin in dark-adapted or 20%-photoisomerized discs in the absence of GTP. **f** Schematic picture of IgG-crosslinked rhodopsin. Fluorescent Fab′-1D4 was rendered bivalent with anti-mouse-*κ*-light-chain antibody IgG. **g** *D*_state_ and occupancies in HMM of IgG-crosslinked rhodopsin in dark-adapted discs (upper) and 20 mM methyl-*β*-cyclodextrin-treated discs (lower). **h** Bar graph of *D*_state_ (left) and stacked bar graph of occupancies (right) of IgG-crosslinked rhodopsin, indicating mean ± SD (*N* = 2). **i** Schematic diagram of rhodopsin dimer light-dependently stabilized by binding with fluorescently labelled transducin (*G*_t_). **j** *D*_state_ and occupancies in three-state HMM of fluorescent Gα_t_ in dark-adapted discs (upper) and 20%-photoisomerized discs (lower) in the presence (hatched bar) or absence (solid bar) of 0.5 mM GTP. Coloured bars are indicated in corresponding *D*_100 ms_-histograms. No optimal HMM was determined for activated-Gα_t_. **k** Bar graph of *D*_state_ (left) and stacked bar graph of occupancies (right) of Gα_t_ in various conditions

Next we examined the effect of light and GTP on the HMM of rhodopsin. Under all tested conditions, the optimal HMM had three diffusive states ostensibly exhibiting invariant diffusion coefficients (Fig. 2a, b). In the presence of GTP, photoisomerization of 20% of the rhodopsin caused no alteration in the HMM (Fig. 2a, b). However, in the absence of GTP, light increased the occupancy of diffusive state-1 (Fig. 2a, b). Correspondingly, light decreased the median *D*_100 ms_ by, at most, about 45%, as a macroscopic study revealed^22^, and reached its minimum with ~20% photoisomerization of rhodopsin (Fig. 2c). Given the 10:1 rhodopsin:G_t_ ratio in discs^40^, this result is consistent with our premise that the binding partner for G_t_ is a rhodopsin dimer^8^. The essentiality of G_t_ and cholesterol for the light-dependent decrease in the *D*_100 ms_ of rhodopsin, in the absence of GTP, was confirmed by depletion and replenishment of G_t_ and/or cholesterol in dark-adapted disc membrane (Supplementary Fig. 4). On the other hand, vbSPT results revealed that the light-dependent hindrance of rhodopsin diffusion is brought about by the increased occupancy of diffusive state-1 (Fig. 2d), by increasing its dwell time (Supplementary Fig. 3a), not by changing the diffusion coefficient of each of the diffusive states.

### Effect of cholesterol-depletion on HMM of rhodopsin diffusion

We previously found that the binding of G_t_ to Rh* stabilizes the rhodopsin dimer to make a highly raftophilic Rh*_2_–G_t_ complex^8^. Thus, it is tempting to speculate that Rh*_2_–G_t_ works like a condensation nucleus in the metastable disc membrane, cooperatively condensing raftophilic metastable rhodopsin dimers, cholesterol and saturated phospholipids. To test this assumption, we investigated how cholesterol participates in the diffusion of rhodopsin in the disc. That was accomplished by examining the effect of cholesterol-depletion, with methyl-β-cyclodextrin (MCD), on the optimal HMM of rhodopsin in dark-adapted or light-exposed discs (Fig. 2e). First, we found that the light-dependently increased state-1 occupancy seen in the absence of GTP can be restored to the level in the dark using 20 mM MCD. This result suggests that the light-dependently formed Rh*_2_–G_t_ can organize a ‘receptor-cluster raft‘. In contrast, MCD did not affect the HMM of rhodopsin in dark-adapted discs, i.e. the slowest diffusive state (state-1) in HMM showed robust tolerance to cholesterol-depletion. Thus, it is highly likely that, even in darkness, rhodopsin molecules autonomously form stable rhodopsin-cluster rafts through protein−protein interactions in highly crowded discs. This presumably happens with the assistance of raftophilic disc lipids other than cholesterol, i.e. the saturated phospholipids such as di-C16:0-phosphatidylcholine in the discs^41^.

### Effect of stabilized-dimerization on rhodopsin diffusivity

To corroborate our assumption that the stabilized-rhodopsin dimer works like a condensation nucleus for rhodopsin-cluster rafts, even in darkness, we examined the diffusivity of IgG-crosslinked rhodopsin (Fig. 2f), which is known to be highly raftophilic^8^. The IgG-crosslinked rhodopsin showed an almost identical three-state HMM to that of rhodopsin when it is light-dependently hindered (compare Fig. 2g upper with 2a lower-middle panel). Cholesterol-depletion restored the HMM to the state-2-dominant one, very much like that in darkness (Fig. 2g lower and 2h).

### The diffusivity of G_t_, Rh_2_*–G_t_ and Gα_t_

The slow diffusion of the Rh*_2_–G_t_ complex was also confirmed by using fluorescently labelled Gα_t_ intact in its light- and GTP-dependent activation (Fig. 2i and Supplementary Fig. 1). The *D*_100 ms_ histogram of Gα_t_ in darkness was almost identical to that of rhodopsin, and showed the light-dependent decrease and activation-dependent increase of Gα_t_ diffusivity (Fig. 2j), in good agreement with a macroscopic study^42^. Furthermore, the optimal HMM of Gα_t_ in darkness (in the form of G_t_) was a three-state model very similar to that of rhodopsin, irrespective of the presence or absence of GTP (Fig. 2j upper and 2k), suggesting pre-coupling^43^ of G_t_ with dark-state rhodopsin. In the absence of GTP, photoisomerization of 20% of rhodopsin reduced the *D*_100 ms_ of G_t_ by increasing the occupancy of diffusive state-1 of G_t_ (Fig. 2j lower and 2k). The vbSPT did not generate any steady HMM for the activated-Gα_t_, presumably owing to the complicated fate of activated-Gα_t_ and its rapid dissociation from the disc membrane^42^.

### Collective behaviour of rhodopsin in dark-adapted discs

**Fig. 3:**
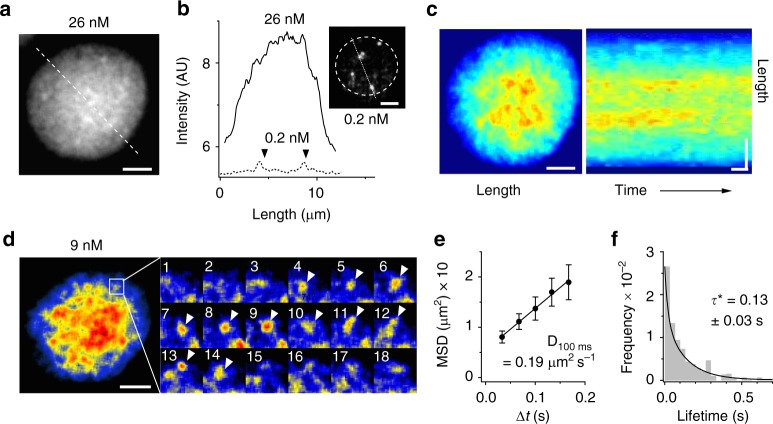
Collective behaviour of rhodopsin in disc membrane. **a** Representative snapshot of fluorescence imaging of a dark-adapted disc incubated with 26 nM fluorescent Fab′-1D4. Scale bar: 2 μm. **b** Fluorescence intensity profiles along the transverse line on a disc membrane and on the dashed line in a representative snapshot of single fluorescent spots of Fab′-1D4 (inset). Arrowheads indicate single-molecule fluorescence spots. **c** Kymographic presentation of dynamic heterogeneity of rhodopsin in a disc membrane. Left: pseudocolour image of a disc membrane incubated with ~20 nM fluorescent Fab′-1D4. The first frame of a movie is shown. Right: a kymograph obtained by three-dimensional-projection of a 1-s movie on ImageJ. Spatial and temporal scale bars: 2 μm and 0.1 s, respectively. **d** Tracking of a solitary cluster of rhodopsin. Left: a pseudocoloured snapshot of a disc membrane incubated with 9 nM fluorescent Fab′-1D4. Scale bar: 2 μm. Right: montage of consecutive images (#1–18) from movie of the square area of disc membrane (1 μm × 1 μm) in the left panel. Arrowheads indicate a transient cluster. Frame rate: 30 s^−1^. **e** Mean-square-displacement (MSD) vs. time interval (Δt) plot of rhodopsin clusters and estimated diffusion coefficient *D* (median ± SE; *N* = 18). **f** Histogram of cluster lifetimes. Lifetime (τ*) was obtained by exponential fitting

To further confirm the formation of rhodopsin-cluster rafts, we attempted to observe rhodopsin-clusters in dark-adapted disc membrane using semi-multimolecule fluorescence imaging. For that, we probed rhodopsin with an approximately two-orders-of-magnitude higher concentration of fluorescently labelled Fab′–1D4 (Fig. 3). Surprisingly, this imaging allowed us to observe apparently uneven (Fig. 3a), vigorously fluctuating (Supplementary Movie 2) fluorescent spots much brighter than the single fluorescent spots. If we assume that the fluorescence intensity of a spot is proportional to the number of rhodopsin molecules forming the spot, then a bright spot can be ascribed to a rhodopsin-cluster. The fluorescence intensity profile along a line traversing the disc membrane shows a convex shape with many indentations (Fig. 3b), suggesting that rhodopsin-cluster rafts are loosely condensed in the central region of dark-adapted disc membranes. Light caused no detectable change in the centrally confined distribution of rhodopsin. Kymographic analysis of rhodopsin-clusters indicated that the cluster lifetime was in the sub-second range, and brighter clusters were concentrated into the disc central area (Fig. 3c). We were able to track the generation and extinction of solitary rhodopsin-clusters (Fig. 3d). The lifetime (*τ*) and diffusion coefficient (*D*) of such clusters were in good agreement with those of diffusive state-1 in the HMM of rhodopsin in darkness (Fig. 3e, f), confirming that the diffusive state-1 is the transient cluster of rhodopsin.

### Involvement of cholesterol in disc inhomogeneity

**Fig. 4:**
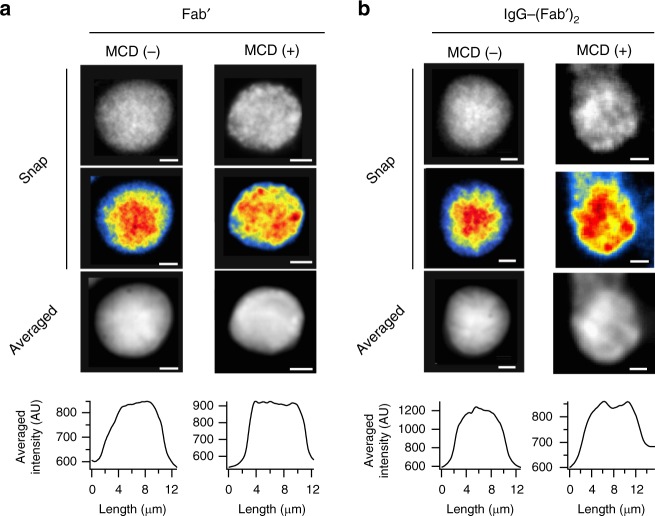
Effect of cholesterol-depletion and IgG-crosslinking on the distribution of rhodopsin. **a** Representative semi-multimolecule fluorescence images indicating the distribution of rhodopsin in discs treated with and without 20 mM MCD (right and left, respectively). Top and upper-middle panels are the still images from a movie, presented in grey-scale and pseudocolour (modified-Jet LUT), respectively. Lower-middle image was obtained by averaging 10 s of the movie. Bottom is the fluorescence intensity profile along the transverse line of the averaged image. **b** Representative semi-multimolecule fluorescence images indicating the distribution of IgG-crosslinked rhodopsin in a disc treated with and without 20 mM MCD (right and left, respectively). The panels are presented in the same sequence as in (**a**). Scale bars: 2 μm

To explore the involvement of cholesterol in the clustering and distribution of rhodopsin in dark-adapted discs, we tested the effect of cholesterol-depletion. Cholesterol-depletion by 20 mM MCD flattened the distribution of rhodopsin-clusters but did not inhibit clustering itself (Fig. 4a). IgG-crosslinked rhodopsin, which is characterized by is high raftophilicity, showed cholesterol-dependent central confinement (Fig. 4b), corroborating our premise that rhodopsin-cluster rafts are segregated into the disc central area through cholesterol-mediated raftophilicity-based interactions.

### Collective behaviour of G_t_ in dark-adapted discs

**Fig. 5:**
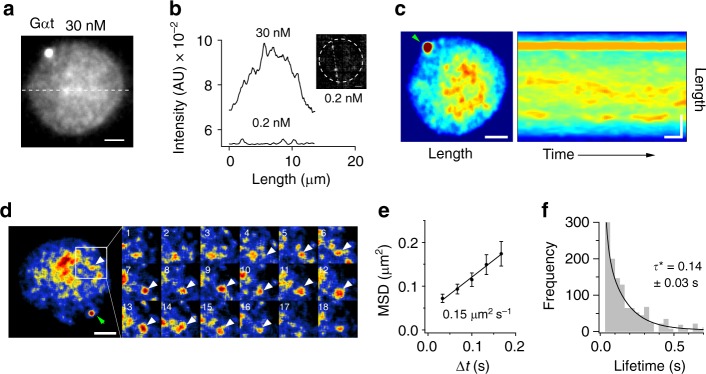
Collective behaviour of transducin in disc membrane. **a** A representative snapshot of fluorescence imaging of a dark-adapted disc incubated with 30 nM fluorescently labelled Gα_t_. Scale bar: 2 µm. **b** Fluorescence intensity profiles along the transverse line on panel (**a**), and on the dashed line in a representative snapshot of the single-molecule fluorescent spots of Gα_t_ (inset). **c** Kymographic presentation of dynamic heterogeneity of Gα_t_ distribution in a disc membrane. Left: pseudocolour image of a disc membrane incubated with ~20 nM fluorescent Gα_t_. The first frame of a movie is shown. Right: a kymograph obtained by three-dimensional-projection of a 1-s movie. Spatial and temporal scale bars: 2 μm and 0.1 s, respectively. **d** Tracking of a solitary cluster of Gα_t_. Left: a snapshot of a disc membrane incubated with 9 nM fluorescent Gα_t_. Scale bar: 2 μm. Right: montage of the portion of the disc membrane (2.8 μm × 2.8 μm) indicated by the open square in left panel. Arrowheads indicate a transient cluster. Frame rate: 30 s^−1^. **e** Mean-square-displacement (MSD) vs. time interval (Δt) plot of Rh clusters and estimated diffusion coefficient *D* (median ± SE; *N* = 18). **f** Histogram of cluster lifetimes. Lifetime (τ*) was obtained by exponential fitting

We also confirmed the clustering of rhodopsin molecules, which are presumably pre-associated with G_t_ in dark-adapted discs, by semi-multimolecule fluorescence imaging of fluorescently labelled Gα_t_ (Fig. 5). The Gα_t_ exhibited central confinement and transient clustering almost exactly like rhodopsin, corroborating our hypothesis. It is also interesting that an aggregate of fluorescently labelled Gα_t_ was often observed at a point on the disc periphery, which is presumably ascribable to the axoneme.

### Concentric heterogeneity in disc membrane

**Fig. 6:**
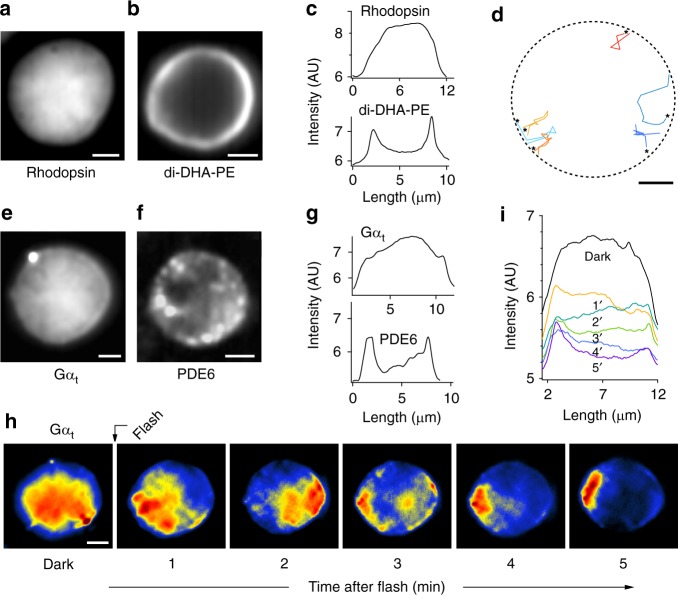
Concentric heterogeneity in disc membrane. **a** Averaged picture (from 10-s movie) indicating rhodopsin distribution in disc membrane incubated with ~20 nM fluorescent Fab′-1D4. **b** Averaged picture (from 10-s movie) showing the annular distribution of fluorescent di-DHA-phosphatidylethanolamine (di-DHA-PE) in disc membrane. **c** Fluorescence intensity profile along transverse lines in panels (**a**) and (**b**). **d** Single-molecule trajectories of di-DHA-PE molecules that jumped into the disc central area, as seen in a representative 2-s movie. Steps in trajectories are colour-coded with time from red to blue. *: start points of trajectories. Dashed line indicates the disc rim. **e** Averaged picture (from 10-s movie) showing loosely, but centrally, confined distribution of Gα_t_ in dark-adapted disc membrane incubated with ~30 nM fluorescent Gα_t_ in the absence of GTP. The bright spot on the upper-left is likely the aggregate of fluorescent Gα_t_ at the axoneme. **f** Averaged picture (from 10-s movie) displaying the marginal distribution of PDE6 in disc membrane incubated with ~30 nM fluorescent anti-PDE6 antibody Fab′-fragment. **g** Fluorescence intensity profile along transverse lines in panels (**e**) and (**f**). **h** A montage of averaged pictures indicating lateral translocation of Gα_t_ upon light-dependent activation. Dark-adapted disc membrane incubated in the presence of 500 µM of GTP was stimulated with a light flash isomerizing 5% of rhodopsin at the indicated time point, and 10-s movies were taken at 1-min intervals. Representative data are presented. **i** Temporal change in fluorescence intensity profile along transverse lines in each picture in (**h**). Scale bars: 2 μm

Our results above implied that disc membranes are segregated into two concentric regions, i.e. a raftophilic centre with confined rhodopsin-clusters, and a non-raftophilic (raftophobic) periphery. To test whether the disc periphery is raftophobic, we examined the distribution of a representative raftophobic phospholipid in the disc membrane, i.e. di-C22:6n3-phosphatidylethanolamine^41^: di-DHA-PE (Supplementary Figs. 1 and 5), and compared it with the distribution of rhodopsin (Fig. 6a, b). Unlike the central confinement of rhodopsin, the raftophobic phospholipid exhibited a vigorously fluctuating annular distribution at the edge of the disc membrane (Fig. 6a−c and Supplementary Movie 3). Furthermore, the single-molecule trajectories showed that PE molecules that jumped from the rim to the disc central area always returned to the rim quickly (Fig. 6d and Supplementary Movie 4), suggesting that the disc central area has the potential for deterring raftophobic PE.

In accordance with the central confinement of rhodopsin, our semi-multimolecule fluorescence imaging of G_t_ revealed that G_t_ is also biased to the disc centre in darkness (Figs. 5a–d, 6e, g and Supplementary Movie 5). In contrast, most of the effector enzyme PDE6 formed immobile hot spots at the disc periphery, and a portion of PDE6 exhibited very fast diffusion (Fig. 6f, g, Supplementary Figs. 1 and 6, and Supplementary Movie 6). This complicated behaviour of PDE6 is consistent with a previous report^44^. Light caused no change in the distribution of PDE6 in our experimental conditions (Supplementary Fig. 6), whereas Gα_t_ showed lateral translocation from centre to periphery during dissociation from the membrane (Fig. 6h, i).

## Discussion

In Supplementary Fig. 7, we present a schematic drawing of the conceptually novel dynamic states of the phototransduction system within the disc membrane. Rhodopsin exists in dynamic equilibrium between three diffusive states presumably ascribable to the cluster raft, dimer and monomer. The slowest diffusive state, accounting for ~20% of rhodopsin in the dark, can be assigned to the rhodopsin-cluster raft. Assuming that the rhodopsin-cluster and the dimer are diffusing in a homogenous lipid membrane (membrane and aqueous viscosities of 8 and 0.2 Poise^22^, respectively) and that diffusive state-2 is the dimer of 2-nm radius, we estimated the size of a rhodopsin-cluster to be ~90 nm in radius, by using an extended-version Saffman−Delbrück equation^45^ (Supplementary Fig. 8). Nevertheless, the large diffusion coefficient of state-3 cannot be explained by the size reduction upon de-dimerization (Supplementary Fig. 8). We may need to assume that the disc membrane has lipid-rich areas differentiated from rhodopsin-dense domains as observed with cryo-electron tomography^46^.

Although it has been shown that rhodopsin can form dimers or oligomers with a lifetime estimated to be on the order of 10 µs^17,25^ via protein−protein interactions, our data revealed that the lifetime of a rhodopsin-cluster is on the order of 100 ms (Figs. 1g and 3f and Supplementary Fig. 3a). Considering our results in this paper and previous findings, the high concentration of rhodopsin that tends to self-organize, the dimerization-dependent raftophilicity of rhodopsin, and the coexistence of raftophilic and extremely raftophobic phospholipids, etc. highly likely confer appropriate stability to rhodopsin-clusters.

In addition, it is intriguing that our vbSPT results indicate that the size of the rhodopsin-cluster is kept constant. We conceive that a delicate balance between protein−protein interactions and the raftophilicity-based stabilizing/de-stabilizing effect of surrounding lipids determines both the lifetime and the size of rhodopsin-clusters. Although molecular interactions that determine the growth, size and stability of receptor-cluster rafts are not fully understood, several key factors are thought to be involved^4,47^. Receptor proteins having raftophilic moiety (e.g. GPI-AR) show oligomerization-induced raftophilicity and form receptor-cluster rafts. The receptor clusters that attain raftophilicity can assemble raftophilic lipids, i.e. cholesterol and lipids with saturated alkyl chains. The long, saturated alkyl chains that are in contact with cholesterol tend to be extended through trans-Gauche isomerization of each methylene segment^4,47^. This alkyl-chain-stretching creates a hydrophobic length-mismatch, relative to the surrounding bulk lipids, driving segregation of receptor-cluster rafts out of the unsaturated bulk lipid membrane phase^4,48^. Coupled with the tendency for cholesterol to be segregated away from the bulk domain, the line tension of the boundary promotes the assembly of receptor-clusters and increases their size^49^. Suppression of the chemical activity of lipids sandwiched within the cluster could also be responsible for stabilizing the cluster^4^. Conversely, the line tension at the interface between the domains of saturated and unsaturated lipids can be reduced by hybrid lipids^50^ (so-called linactant^51^ or 2D-detergent^52^), where one chain is saturated and the other unsaturated. This can allow finite-size domains to be stable even in equilibrium^50^. These situations should coincide with that of the rhodopsin-cluster raft. However, the hybrid lipids in the disc membrane account for ~65% of total lipids and having polyunsaturated fatty acid like DHA^53^ at the β-position. Therefore, they may destabilize the rhodopsin-cluster rafts by their solubilizing ability. In sum, the lifetime and the size of rhodopsin-cluster is likely determined by a delicate balance of these reciprocal factors, although the exact mechanism remains to be determined. In connection with this, we have previously shown that the dimerization-dependent raftophilicity of rhodopsin essentially requires palmitoyl modifications of rhodopsin. Thus, we had intended to examine the role of palmitoyls in rhodopsin diffusion and distribution. However, in this study, we could not address these issues, because the reducing agents, which break up palmitoyl-cysteine thioester linkages, caused deleterious effects on the shape of f-ROSs.

Given our result that the rhodopsin-clusters in dark-adapted discs tolerate 20 mM MCD, the raftophilic lipids that mainly contribute to the construction of rhodopsin-cluster rafts may not be cholesterol, but, rather, raftophilic saturated-phospholipids such as di-saturated phosphatidylcholine, the main raftophilic phospholipid in the disc^41,53^. Alternatively, the tolerance of 20 mM MCD may be ascribable to a particular state of cholesterol in the rhodopsin-cluster raft, whereby cholesterol is shielded from the MCD. In fact, about 20% of the cholesterol remains in the disc membrane after 20 mM MCD treatment (Supplementary Fig. 9). Collapse of f-ROSs with higher concentrations of MCD hampered our exploration into the essentiality of the presumably small amount of cholesterol in rhodopsin clustering.

The optimal HMM of rhodopsin in the darkness, in which the medium diffusive state (S2)-dominant, suggests that only a limited amount of rhodopsin forms clusters and that the many remaining rhodopsin molecules are in the dimeric or monomeric state. Such a distribution may be due to the lipid composition of the disc membrane. The raftophilic phospholipid component in the disc membrane is di-saturated phospholipid (mainly 16:0–16:0 phosphatidylcholine^53^), accounting for only 8% of total phospholipid^41^. Thus, the deficiency of raftophilic phospholipids may prevent the incorporation of all rhodopsin into clusters. In this context, diffusive state-2, accounting for 50% of rhodopsin, must be ascribable to metastable rhodopsin dimers that have not been stabilized by such raftophilic lipids, instead, probably being accommodated in the hybrid lipids that account for ~65% of total phospholipid in the disc membrane^41^.

Finally, state-3 might be monomeric rhodopsin, because an appreciable amount of rhodopsin is estimated to exist as monomers^26^, and we observed an extremely large diffusion coefficient, as in state-3, for rhodopsin incorporated in the supported-planar bilayer (Supplementary Fig. 10).

Meanwhile, the high diffusivity of G_t_- or IgG-bound rhodopsin in state-3 of the optimal HMM is puzzling. However, it should be noted that IgG-crosslinking does not mean complete fixation of two rhodopsin molecules. Instead, IgG would contribute to stabilizing the rhodopsin dimer by reducing the free volume in the membrane of two rhodopsins via binding to the tips of the C-terminal peptides extended ~5 nm into the cytoplasm. If that is the case, the lipid environment that can destabilize the rhodopsin dimer would cancel the raftophilicity of IgG-crosslinked rhodopsin. Thus, the highly diffusive state seen in HMM of IgG-crosslinked rhodopsin suggests the presence of a region in which rhodopsin dimerization is hindered in the disc membrane. The low-density regions in the disc membrane observed with cryo-electron tomography may coincide with this region^46^. It is also indisputable that the lipid environment may affect the G_t_-stabilized rhodopsin-dimerization.

What is the physiological significance of the dynamic non-uniformity of the disc membrane brought about by generation and extinction of rhodopsin-cluster rafts? Taken together, our results support a hypothesis that receptor-cluster rafts likely contribute to achieving the high efficacy of phototransduction. Logically, transient rhodopsin-cluster rafts do not contradict the hypothesis that the array of rhodopsin dimers acts as the ‘signalling scaffold’^45^ or “kinetic trap”^5^ responsible for single-photon detection^10,43^. Pre-associated G_t_ with a high dissociation rate is expected to quickly scan the array to find Rh*. The lifetime of the rhodopsin-cluster raft (on the order of 100 ms) is sufficiently long compared with the time required for G_t_ activation by Rh* (~4 ms)^54^. A simulation study proposed that longer cluster lifetime and larger cluster size aids higher G_t_-activation-efficacy in single-photon regimes^10^. Actually, classical electrophysiological studies have shown that the efficiency of photon capture and conversion into an electrical signal by rods increases with decreasing temperature^9^, by which rhodopsin-cluster rafts could be stabilized. Consistent with this, we found that the diffusive state-1 lifetime increases at a lower temperature (Supplementary Fig. 3). In addition, the raftophilicity of supramolecular structures of rhodopsin can contribute to efficient phototransduction by increasing the lifetime of Rh*, by recruiting a Ca^2+^-dependent inhibitor of rhodopsin kinase, i.e. recoverin, which has a higher efficacy in the raftophilic environment^55^. It is also known that the base of the ROS contains more cholesterol than does the tip^56^, so the base should be useful for forming rhodopsin-cluster rafts. This hypothesis is consistent with a recent finding that the amplitudes of saturating and single-photon responses decreased by 5–10 times when illumination of the tip of the ROS is compared with that of the base^57^.

Regarding the supramolecular structure of rhodopsin, to which some G_t_ weakly pre-associates in the dark, a single photon is expected to be able to activate all of the G_t_ molecules on the supramolecular structure of rhodopsin in a short period of time^10,43^. Therefore, the invariance in the size of rhodopsin-cluster suggested in our experiment may be able to explain the constancy in the amplitude of the single-photon response^16^ of rod-photoreceptors. This kind of digital-like signalling has also been observed in GPI-AR signalling in cell membrane^58^. In this regard, the constant-sized transient rhodopsin-cluster rafts may have a role in providing inherently distributed and stochastic platforms that can generate a quite-uniform digital-like response to single photons in rod photoreceptors. This mechanism would also be able to respond to a stronger photic stimulus simply by the summation of digital-like responses.

In addition to its potential importance in phototransduction, the dimerization-dependent raftophilicity of rhodopsin, with resultant raftophilic cluster formation and concentric disc inhomogeneity, may have particular importance in disc morphogenesis and maintenance of rod photoreceptors. It was recently demonstrated that three enigmatic mutants of rhodopsin, known to cause the blinding disease retinitis pigmentosa, inhibit rhodopsin dimerization^59^. This suggests that, as the nucleating step of rhodopsin self-organization may not occur efficiently in such discs, the supramolecular organization of rhodopsin is compromised with severe consequences for disc architecture and stability.

Our data on the confinement of rhodopsin into the disc central area, and the ring-like distribution of PE in the disc peripheral area implicate the concentric inhomogeneity of the disc membrane in terms of raftophilicity. We believe that such concentric segregation is based on the hydrophobic mismatch between the hydorophobic length of rhodopsin-cluster raft and the hydrophobic thickness of the lipid bilayer lining the disc rim. Each disc is bounded by a rim comprised of tetraspanin complex peripherin-2/rds–Rom-1^60^, where the membrane is distorted into an energetically unfavourable high-curvature bend. It has recently shown that the intrinsically disordered cytoplasmic C-terminus of the peripherin-2/rds can generate membrane curvature by associating with cone-shaped lipids such as PE having a small polar head group and bulky polyunsaturated acyl chains^61^. Thus, it is conceivable that the rim protein complexes provide a thin framework for the lipid bilayer at the edge of the lamellar region of the disc by recruiting raftophobic phospholipids that reduce the hydrophobic thickness of the bilayer, e.g. di-DHA-PE would make a bilayer of ~25 Å in hydrophobic thickness^62^. If this is the case, rhodopsin having ~27 Å^63^ in hydrophobic length and the rhodopsin-cluster, which would have a longer hydrophobic length than rhodopsin, would be excluded from the disc periphery. In support of our hypothesis, AFM shows a protein-free lipid bilayer girdle between the disc centre, filled with nanodomains of rhodopsin, and the disc rim in a disc membrane fixed on a mica surface^13^.

In summary, our single and semi-multimolecule results show that rhodopsin autonomously forms a sort of receptor-cluster rafts, which are in dynamic equilibrium with the lower oligomeric-states of rhodopsin in the disc membrane. Stochastic rhodopsin-cluster rafts provide meso-sized raftophilic signalling platform that is highly likely responsible for the single-photon detection in rod-photoreceptors. Further, the coexistence of multimodal forms of rhodopsin in dynamic equilibrium may allow receptor signalling to have a wide dynamic range, flexibility, and homeostasis of phototransduction machinery. In addition, the concentric heterogeneity in raftophilicity in the disc membrane may play important roles not only in the regulation of phototransduction but also in maintaining homeostasis of photoreceptor. Together with previously accumulated evidence, our results imply that both the constitutive and the stimulation-dependent clustering of rhodopsin-like GPCRs may have important roles not only in signalling, but also in the biogenesis and maintenance of membrane architecture in the cell, based on their receptor-cluster-raft organizing ability.

## Methods

### Ethics statement

This study was approved by the Institutional Animal Care and Use Committee, and carried out according to Kobe University’s animal experimentation regulations.

### Materials

Monoclonal antibody (1D4) against the carboxyl terminus 9-mer peptide of rhodopsin^64^ was purchased from the University of British Columbia (Vancouver, Canada) via Flintbox. Antibody against PDE6 was prepared by immunizing rabbits with a peptide corresponding to the apical end of the PDE6a of *Rana pipiens*, i.e. ^137^Asp-^156^Val: accession number AAK95399 (See Supplementary Fig. 1). HiLyte Fluor 750 -C2–maleimide (HL750-maleimide) and HL750-*N*-hydroxysuccinimide ester (HL750-NHS) were purchased from Ana Spec Inc. (Fremont, CA). Monoclonal antibody to mouse IgG-κ-light-chain was purchased from Yamasa Corporation (Chiba, Japan). Urea-treated rod outer segment (ROS) membranes, G_t_ and Gβγ_t_ were prepared from frog ROS membranes as described previously^65^. di-DHA-PE (1,2-didocosahexaenoyl-sn-glycero-3-phosphatidylethanolamine (PE)), DiynePC (1,2-bis(10,12-tricosadiynoyl)-sn-glycero-3-phosphocholine), DOPC (1,2-dioleoyl-sn-glycero-3-phosphocholine), DPPC (1,2-dipal- mitoyl-sn-glycero-3-phosphocholine), Chol (cholesterol (ovine wool)), GM1 Ganglioside (bovine brain) and Rho-PE (1,2-dioleoyl-sn-glycero-3-phosphoethanolamine-N-lissamine rhodamine B) were purchased from Avanti Polar Lipids (Alabaster, AL). Cholera toxin B-Alexa Fluor 488 conjugate (CTB488) was purchased from Molecular Probes (Eugene, OR). Bullfrogs (*Rana catesbeiana*) were purchased from Mr. Kazuo Ohuchi (Saitama, Japan). Other standard chemicals were mainly purchased from Merck (Kenilworth, NJ), Fuji Film Wako Pure Chemical (Osaka, Japan), and Dojindo Molecular Technologies, Inc. (Kumamoto, Japan). Protease-inhibiting peptides were purchased from Peptide Institute Inc. (Osaka, Japan). Precast sodium dodecyl sulfate (SDS)-polyacrylamide gels (XV-Pantera gel; 5–20%) were purchased from DRC Co. Ltd (Tokyo, Japan).

### Buffers

Standard buffers contained (in mM, unless stated otherwise): buffer A—KCl 60, NaCl 30, MgCl_2_ 5, 3-morpholinopropanesulphonic acid 10, phenylmethylsulfonyl fluoride 0.2, aprotinin 5 µg ml^−1^, E64 10 µg ml^−1^ (pH 7.5 at 0 °C); buffer B—Tris-HCl (pH 7.5) 10, 1,4-dithio-DL-threitol (DTT) 5, MgSO_4_ 6, EDTA 1, MCD 5, and 25% glycerol; buffer C (Ringer’s solution)—NaCl 110, KCl 2.5, MgCl_2_ 2, CaCl_2_ 1, glucose 1, 2-[4-(2-Hydroxyethyl)-1-piperazinyl]-ethanesulfonic acid (HEPES)-HCl 10 [pH 7.5]; buffer D—k-gluconate 115, KCl 2.5, MgCl_2_ 2.5, BAPTA 0.1, CaCl_2_ 0.01, HEPES-KOH (pH 7.45) 10, taurine 0.2, aprotinin 2 µg ml^−1^, E64 5 µg ml^−1^ and leupeptin 0.005.

### Preparation of frog rod outer segment (ROS) membranes

ROS membranes of bullfrog (*Rana catesbeiana*) were prepared as described previously^28^ and stored at −80 °C in the darkness.

### Fluorescent labelling of Fab′-1D4 against the C-terminus of rhodopsin and Fab′ of an antibody against frog PDE6

Epitopes for antibodies against rhodopsin and PDE6 are illustrated in Supplementary Fig. 1a. The Fab′ fragments were labelled with HL750-C2 maleimide as described previously^66^, and purified by Superose 12 (3.2 × 300) column chromatography on a SMAT system (Pharmacia; Uppsala, Sweden). The dye:protein ratio was ~1.

### Fluorescent labelling of Gα_t_

The Gα_t_ was directly labelled with HiLyte Fluor 750-C2 maleimide. The ROS membrane containing 300 nano-mole (~10 mg) of rhodopsin was exposed to room light at 0 °C for 20 min and spun down by ultracentrifugation (100,000 × *g*, 5 min). Membranes were resuspended with 30 ml of buffer A containing 1 mM methyl-β-cyclodextrin (MCD) and 5 mM DTT and pelleted by ultracentrifugation (170,000 × *g*, 15 min). This washing procedure was repeated three times. During this procedure, peripheral membrane proteins including rhodopsin kinase (GRK1) were removed^67^. Then, membranes were suspended and centrifugally washed three times with 30 ml of buffer A containing 20 mM MCD and 5 mM DTT, to remove cGMP-phosphodiesterase 6 (PDE6) and excess Gβγ_t_^67^. Resulting membranes were washed with 20 ml of buffer A containing 0.2 mM Tris(2-carboxyethyl)phosphine-HCl (TCEP) for three times in the same manner as above. Finally, the membranes were suspended in 5.28 ml of the same buffer at 0 °C, and quickly mixed with 1.19 × 10^−6^ M of HL750-maleimide in 300 µl buffer A (TCEP:dye ratio was 1.125 in final concentration)^68^. The reaction proceeded at 0 °C for 40 min, and was stopped with 25 µl of 1 mM 2-mercaptoethanol. Resulting membranes were washed three times with 20 ml of buffer A containing 1 mM DTT. Finally, HL750-Gα_t_ was extracted by suspending the membranes in 1.8 ml of buffer A containing 1 mM guanosine-5′-triphosphate (GTP), 1 mM DTT and 1 mM MCD, followed by ultracentrifugation (452,000 × *g*, 5 min). This extraction procedure was repeated five times. Pooled extract containing HL750-Gα_t_ was applied to a Blue-Sepharose column (handmade; 1 ml in column volume) equilibrated with buffer B, and trapped HL750-Gα_t_ was eluted with linear concentration gradient of NaCl (0–0.5 M) on Smart System. A single fluorescently labelled band of purified HL750-Gα_t_ in peak fraction was detected by SDS-polyacryl amide gel electrophoresis, using a 750-nm scattered laser beam for excitation and a CCD camera (Hamamatsu Photonics, Hamamatsu, Japan; C9100-12) equipped with a long-pass filter 770ALP (Omega Optical, Brattleboro, VT) and a macro lens (see Supplementary Fig. 1b). HL750-Gα_t_ was stored at −20 °C in 50% glycerol. The dye:protein-ratio of HL750-Gα_t_ was measured by spectrophotometry to be ~0.7 using *ε* = 30,400 M^−1^ cm^−1^ at 280 nm for Gα_t_, and 250,000 M^−1^ cm^−1^ for HiLyte Fluor 750.

### Determining the labelling site on HL750-Gα_t_

HL750-Gα_t_ was subjected to in-gel digestion by Lys-C endoproteinase (Roche, Basel, Switzerland) as described^69^. We used Perfect-NT gels (DRC, Tokyo, Japan; NTH-575HP; 5–20%) for isolation of HL750-Gα_t_, and NuPAGE Novex 12% gels (Invitrogen,) for peptide separation. A single major peptide (MW~5000) labelled with HL750 was transferred to a PVDF membrane (see Supplementary Fig. 1c), and the N-terminal amino acid sequence of the peptide was analysed by a protein sequencer (Applied Biosystems, Carlsbad, CA) and compared with predicted LysC-digestion segments of *Xenopus* Gα_t_ (P38407-1) (see Supplementary Fig. 1a).

### Evaluating intactness of HL750-Gα_t_ in light- and GTP-dependent activation

Functional intactness of HL750-Gα_t_ was confirmed by its activation-dependent release from a reconstituted system comprising HL750-Gα_t_, Gβγ_t_ and urea-treated ROS membrane. Urea-treated ROS membrane containing 50 µg of rhodopsin was incubated with 15 pmole of HL750-Gα_t_ and an equal amount of Gβγ_t_ in 50 µl of buffer A containing 1 mM ATP at 0 °C overnight. The ROS membranes were exposed to light for 10 min or kept in the darkness, in the presence or absence of 500 µM GTP and 500 µM GDP. Then the membranes were spun down by ultracentrifugation at 350,000 × *g* for 5 min. Proteins in aliquots (8 µl) of supernatants were separated in SDS-polyacrylamide gel electrophoresis, and protein bands containing HL750-Gα_t_ were detected by a handmade near-IR imaging apparatus (see Supplementary Fig. 1d).

### Fluorescent labelling of di-DHA-PE

di-DHA-PE was labelled with HL750 SE. 0.6 µM of di-DHA-PE in 50 µl of chloroform was mixed with 2 µl of triethylamine, and then 300 nM of HL750-NHS, dissolved in 5 µl of dimethylsulfoxide, was added. After incubation at room temperature for 2 h, the reaction product was dried by evaporation and dissolved with chloroform:methanol:water (65:25:4). Fluorescently labelled di-DHA-PE was purified on a high-performance thin layer chromatography plate (Merck Millipore, Burlington, MA, #105641) by developing it with chloroform:methanol:NH_4_OH (65:35:8). The blue band on HPTLC was scraped off from the plate, and the PE was extracted from the silica gel by washing three times with 1 ml of chloroform:methanol:water (65:25:4). The extract was lyophilized to dryness, and dissolved with 1.5 ml of chloroform/methanol (2:1). About 48 µM of HL750-di-DHA-PE was obtained, and kept under N_2_ atmosphere at −30 °C.

### Preparation of fragmented ROS

All procedures were performed in complete darkness using IR goggles from NEC (Tokyo, Japan). Intact ROS was prepared from the retinas of dark-adapted bullfrogs by the method described previously^70^. Briefly, each retina with pigment epithelium was gently placed on three-layered filter papers with the pigment epithelium-side upward. After the vitreous body was absorbed by the filter papers, retinas were cut out using scissors, with a back-up sheet, and kept in buffer C. The retinas with filter papers were placed on a paraffin block covered with Parafilm, attached with several pins, and immersed in 0.8 ml of buffer C per retina. ROSs were detached from the retinal surface by agitating with repetitive pipetting of 50-µl aliquots of buffer C through a large-bore pipette tip (Cell Saver Tip PT-003, InaOptica, Osaka, Japan). Crude ROS suspension was overlaid on a step-gradient of Percoll in buffer C, consisting of 0.6 ml of 70%, 0.3 ml of 50%, and 0.6 ml of 26% Percoll in buffer C, and centrifuged at 17,000 × *g* for 2 min at 4 °C. Bands corresponding to intact ROS and to inner segment-attached ROS were harvested, diluted with the same volume of buffer C, and spun down by centrifugation (100 × *g* for 3 min). Pellets were suspended with 0.8 ml of ice-cold buffer D. The intact ROSs were then broken into short fragments, i.e. fragmented-ROS (f-ROS), by passing the suspension through a 27-gauge needle eight times. When we observed fluorescently labelled proteins on the disc membrane, we added the proteins in buffer D containing 1 mg ml^−1^ ovalbumin to the suspension of 400 µl of f-ROS suspension in buffer D of 0.2–30 nM in final concentration. After 1 h of incubation, the sample was overlaid on a Percoll density-gradient consisting of 0.3 ml of 44%, 0.3 ml of 40% and 0.9 ml of 26% Percoll in buffer D, and then centrifuged at 34,000 × *g* for 5 min at 4 °C. The f-ROSs were harvested from the interface between the 44 and 40% Percoll layers. The f-ROS suspension was diluted with two volumes of buffer D and kept at 0 °C in a light-tight container until use. When we applied HL750-di-DHA-PE to f-ROS, 10 µl of labelled-PE was evaporated by N_2_ gas flow and solubilized with 10 µl of methanol. The methanol solution was diluted with 1 ml of buffer D, and a 5-µl aliquot was added to 500 µl of f-ROS suspension containing approximately 3 nM of rhodopsin. After about 3 h of incubation on ice in the dark, samples were used for experiments. To observe the behaviour of IgG-crosslinked rhodopsin in the disc membrane, we employed FL750–Fab′1D4 rendered bivalent via a monoclonal antibody. The IgG-crosslinked HL750–Fab′1D4 was purified by Superose 12-column chromatography and applied to the f-ROS suspension (~20 nM in final concentration).

### Single-molecule imaging

A suspension of f-ROS with fluorescent probe was introduced into a small chamber made of Secure-Seal from GRACE Bio-Labs (Bend, OR) on a glass slide (Matsunami, Tokyo, Japan) placed in an Attofluor Cell Chamber from Invitrogen (Paisley, UK). After 10 min, sedimentation of f-ROS onto the glass surface was complete. The chamber was set on the stage of a total internal reflection fluorescence microscope (TIRFM; Nikon, Tokyo, Japan, TE2000), and the aqueous phase was continuously perfused (0.1 ml min^−1^) with a buffer containing an oxygen-scavenging system freshly prepared by mixing substrate (2.25 mg·ml^−1^ glucose) and enzymes (216 µg ml^−1^ glucose oxidase and 36 µg ml^−1^ catalase). When GTP was applied to f-ROSs, the chamber was perfused with freshly prepared perfusion buffer containing 500 µM of GTP. Fluorescently labelled Fab′1D4 on disc membranes was illuminated with the highly inclined laser beam of 750-nm wavelength. Images were acquired with an electron-multiplying CCD camera C9100-12 (Hamamatsu Photonics, Hamamatsu, Japan) at a spatiotemporal resolution of 30 frames s^−1^ and 76 nm pixel^−1^. The TIRFM was equipped with Nikon 100×/1.45 Plan-Apo objectives. A filter set consisting of 760DRLP (Chroma Technology Corp., Bellows Falls, VT) and HQ810/90 (Omega Optical) was used.

### Single-molecule tracking

Coordinate points of fluorescent spots were measured with TrackMate on Fiji (http://fiji.sc/TrackMate) using a Laplacian of Gaussian detector for segmentation on the image and a Simple Linear Assignment Problem (LAP) tracker for the particle-linking algorithm. Localization error was assessed to be ~50 nm by single-molecule tracking of immobile spots of fluorescently labelled proteins on a glass surface. Splitting and merging events were ignored. Effective diffusion coefficients of single fluorescent molecules within 100 ms (*D*_100 ms_)^39^ were evaluated by mean square displacement (MSD)-time intervals (Δ*t*) using a per-value class on MatLab of MathWorks (Natick, MA), i.e. msdanalyzer (http://tinevez.github.io/msdanalyzer/)^71^. Whole trajectories (minimum trajectory length >15 frames; average trajectory length ~35; average number of trajectories ~400) obtained by TrackMate were imported into msdanalyzer. We eliminated trajectories yielding bent curves, which likely resulted from partial confinement of membrane molecules by incisures of the disc membrane, based on the criterion of good-enough-fitting (*R*^2^ > 0.8) of the MSD-Δ*t* curve (http://tinevez.github.io/msdanalyzer/tutorial/MSDTuto_brownian.html).

### Variational Bayes single-particle tracking (vbSPT) analysis of single-molecule trajectories

Time-series data provided by single-molecule experiments offer the opportunity to infer not only model parameters describing transition rates between molecular states, but also information about the model itself, e.g., the number of molecular states. If the complex of interest transitions from one locally stable diffusive state to another, the experiment is well-modelled by a hidden Markov model (HMM)^35,72^, a probabilistic model in which an observed time series is conditionally dependent on a hidden, or unobserved, discrete state variable. To extract diffusive states and transition rates of rhodopsin diffusing in retinal disc membranes, we applied an open-source software vbSPT (http://www.sourceforge.net/projects/vbspt)^36^ to single-molecule tracking data used in MSD-Δt analysis. The number of iterations and bootstraps were set to 25 and 100, respectively. The vbSPT method uses a maximum-evidence criterion to determine the underlying parameters and the number of diffusive states from the observed data. We validated vbSPT using simulated reaction diffusion trajectories in a disc geometry (a circle 8 µm in diameter) and in a 1 × 2-µm area mimicking a small lobule of frog disc membrane, with the same trajectory length distribution as that of our experimental data and using realistic localization errors (50 nm), diffusion coefficients and transition parameters (Supplementary Table 2). The method successfully recovered the parameters used for simulating the data.

### Semi-multimolecular fluorescence imaging of rhodopsin, Gα_t_ PDE6 and PE on disc membranes

To observe the collective behaviour of rhodopsin, Gα_t_ and PDE6 in disc membranes, we used f-ROSs incubated with approximately 10–30 nM of HL750-Fab′1D4, HL750-labelled Gα_t_, or HL750-Fab′ of anti-PDE6 antibody. Except for using approximately two-orders-of-magnitude higher concentration of fluorescent probes, there were no differences between the semi-multimolecule fluorescence imaging and single-molecule imaging. To observe the collective behaviour of a highly raftophobic cone-shaped phospholipid, PE, we used HL750-di-DHA-PE. An aliquot (10 µl) of fluorescent di-DHA-PE in chloroform:methanol:H_2_O was dried under N_2_ gas flow. The dried material was dissolved in methanol at 60 °C and dispersed into the f-ROS suspension, to be 4.8 nM in final concentration. Following 30 min incubation, we performed semi-multimolecule fluorescence imaging of di-DHA-PE in the disc membranes.

### Determination of *D*_100 ms_ of rhodopsin clusters

Coordinate points of fluorescent spots were measured with TrackMate on Fiji using a Laplacian of Gaussian detector for segmentation on the image and a Simple LAP tracker for the particle-linking algorithm, and manually edited after inspection. *D*_100 ms_ was evaluated by the per-value class “msdanalyzer” on MatLab as described above.

### Photoisomerization of rhodopsin in discs

The suspension of f-ROS in the perfusion chamber was illuminated by a flashlight (Contax TLA140 from Kyocera, Kyoto, Japan) through a green filter #OG515 from Schott AG (Mainz, Germany). The amount of isomerization was determined by the change in the absorbance at 504 nm of f-ROS suspension in the chamber on TIRFM before and after a bright light flash. Then, we obtained arbitrary light intensity by using neutral filters.

### Manipulation of membrane cholesterol content

To deplete cholesterol in the disc membranes for single-molecule tracking experiments, f-ROSs were suspended in buffer A containing 20 mM MCD. Treated f-ROSs were isolated by Percoll density-gradient centrifugation using 44, 40, and 26% step gradients of Percoll as described above. The cholesterol content was decreased by about 80% by the MCD treatment (see Supplementary Fig. 9), as assessed by Amplex Red Cholesterol Assay Kit (Thermo Fisher Scientific, Waltham, MA). To replenish cholesterol, cholesterol-depleted f-ROSs were incubated with buffer A containing 0.6 mM cholesterol and 20 mM MCD for 1 h at 0 °C in complete darkness. Cholesterol-replenished f-ROS was spun down by gentle centrifugation (100 × *g* for 3 min) and resuspended with buffer A. All procedures were performed in complete darkness.

### Determining the diffusion coefficient of monomeric rhodopsin in a fluid lipid bilayer membrane

Rhodopsin was reconstituted into a preformed supported planar bilayer (SPB) membrane, composed of 1-palmitoyl-2-oleoyl-phosphatidylcholine, by the rapid dilution of detergent-solubilized Cy7-labelled rhodopsin^73^. Single-molecule tracking was performed on TIRFM, and obtained trajectories were subjected to MSD analysis to determine *D*_100 ms_.

### Assessment of the raftophobic nature of HL750–di-DHA-PE

The raftophilicity of fluorescently labelled di-DHA-PE was assessed based on its distribution into liquid-disordered (*L*_d_) and liquid-ordered (*L*_o_) phases artificially formed on the SPB membrane^74^. Patterned separation of *L*_o_ and *L*_d_ phases was induced in DOPC:DPPC:Chol (1:1:1) (with GM1 and Rho-PE (1% each)). Ten microliters of 4.8 nM HL750-di-DHA-PE was evaporated by N_2_ gas flow and solubilized with 10 µl of methanol. The methanol solution was diluted with 0.1 ml of buffer D, and a 5-µl aliquot was added to 500 µl of aqueous solution beyond the SPB membrane (see Supplementary Fig. 5).

### Statistics and reproducibility

Statistical analysis was performed using MatLab. Differences between multiple groups were assessed by one-way or two-way analysis of variance (ANOVA), as appropriate, followed by Tukey’s multiple-comparison test. Differences between two histograms were assessed with the Mann−Whitney *U* test.

### Reporting summary

Further information on research design is available in the Nature Research Reporting Summary linked to this article.

## Supplementary information

Supplementary Information

Supplementary Data 1

Reporting Summary

Description of additional supplementary items

Supplementary Movie 1

Supplementary Movie 2

Supplementary Movie 3

Supplementary Movie 4

Supplementary Movie 5

Supplementary Movie 6

## Data Availability

Data that support the findings of this study are available from the corresponding author upon reasonable request. Raw data used to generate the plots can be found in Supplementary Data 1 file accompanying this manuscript.
