# Supplementary material for: Raftophilic rhodopsin-clusters offer stochastic platforms for G protein signalling in retinal discs
Source: Commun Biol. 2019 Jun 14;2:209. doi: 10.1038/s42003-019-0459-6 (PMC6570657; doi:10.1038/s42003-019-0459-6)
Supplement: Supplementary file 1 — Supplementary Information [file 42003_2019_459_MOESM1_ESM.pdf]

# Supplementary Figures

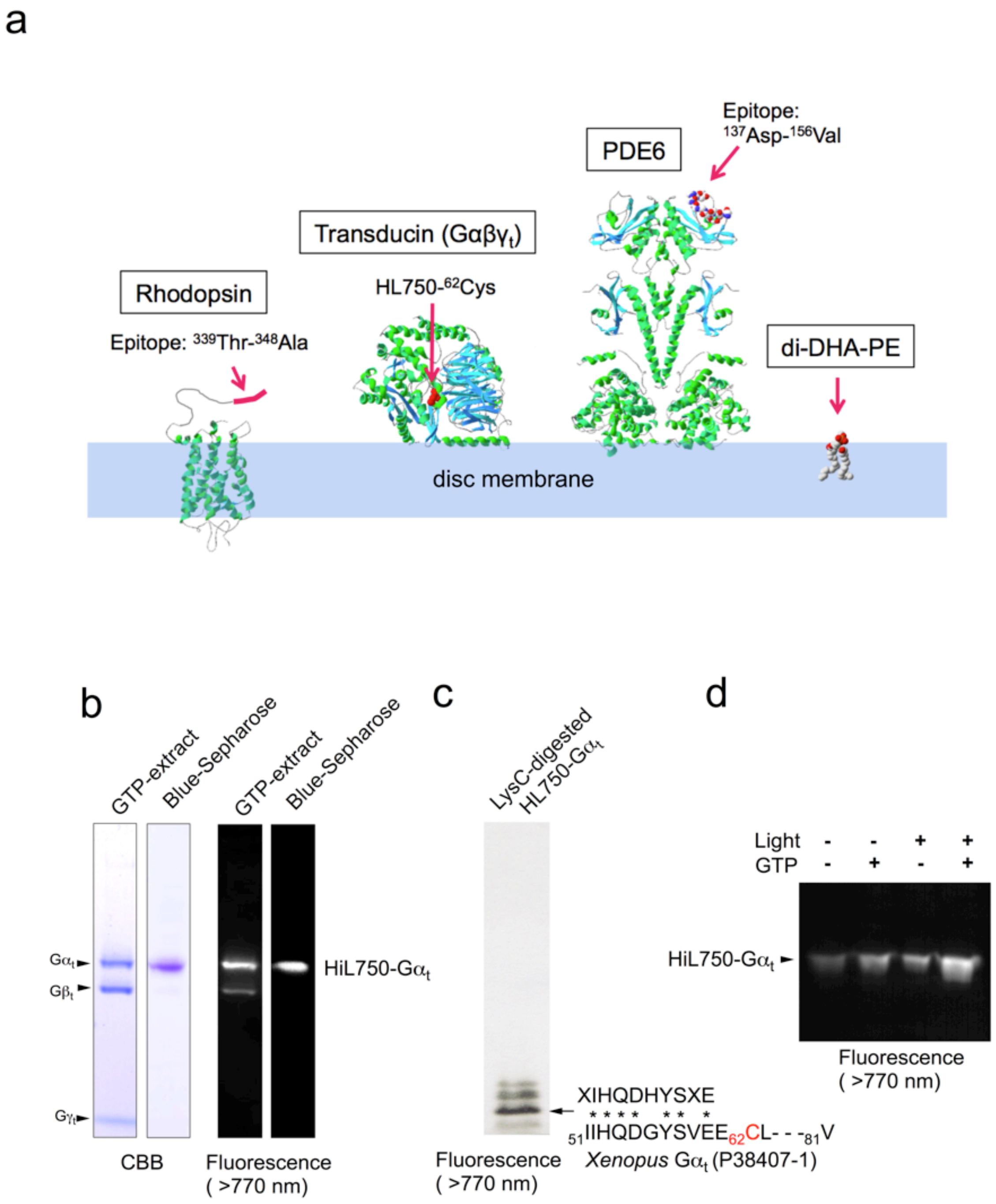


**Supplementary Figure 1 | Fluorescent probes**

**a**, Antibody binding sites on rhodopsin (Rh) and PDE6, and fluorescently labelled sites on $G\alpha_t$ and phosphatidylethanolamine (di-DHA-PE). The epitope of

monoclonal antibody (1D4) against the carboxyl terminus 9 amino acids ($^{339}$Thr-$^{348}$Ala) of mouse Rh (PDB ID:2i37). The fluorescently labelled site on frog G$\alpha_t$ ($^{62}$Cys at the N-terminus of helix-3 in its helical region) is indicated in the heterotrimeric complex of G$\alpha_t$G$\alpha_i$ chimera and G$\beta\gamma_t$ (PDB ID:1GOT). The peptide corresponding to the epitope of antibody against frog PDE6-α, i.e.$^{137}$Asp-$^{156}$Val, on the apical end of the α-subunit of PDE6 (PDB ID:3JBQ). The fluorescently labelled site on phosphatidylethanolamine (di-DHA-PE) is its amine residue. The structure of di-DHA-PE was obtained from PDB ID:1EYS. b, Purification of HL750–G$\alpha_t$. G$\alpha_t$ on light-exposed rod outer segment (ROS) membranes was extracted with GTP and purified by Blue-Sepharose column chromatography. Protein bands were visualized with Coomassie brilliant blue staining, and HL750–labelled bands were visualized by their fluorescence. **c**, Fluorescently labelled cysteine residue determined by in-gel-digestion followed by amino acid sequencing of labelled peptide. Amino acid sequence of a major band showed a high identity with $^{51}$I-$^{81}$V of Xenopus G$\alpha_t$ (UniProtKB/Swiss-Prot: P38407.2) containing $^{62}$C. **d**, Assessment of the intactness of HL750–G$\alpha_t$ in its Rh*- and GTP-dependent activation. HL750–G$\alpha_t$ was reconstituted with G$\beta\gamma_t$ on urea-washed ROS membrane, and activation-dependently released HL750–G$\alpha_t$ from the membrane was separated by SDS-polyacrylamide gel electrophoresis and visualized by fluorescence imaging.

a

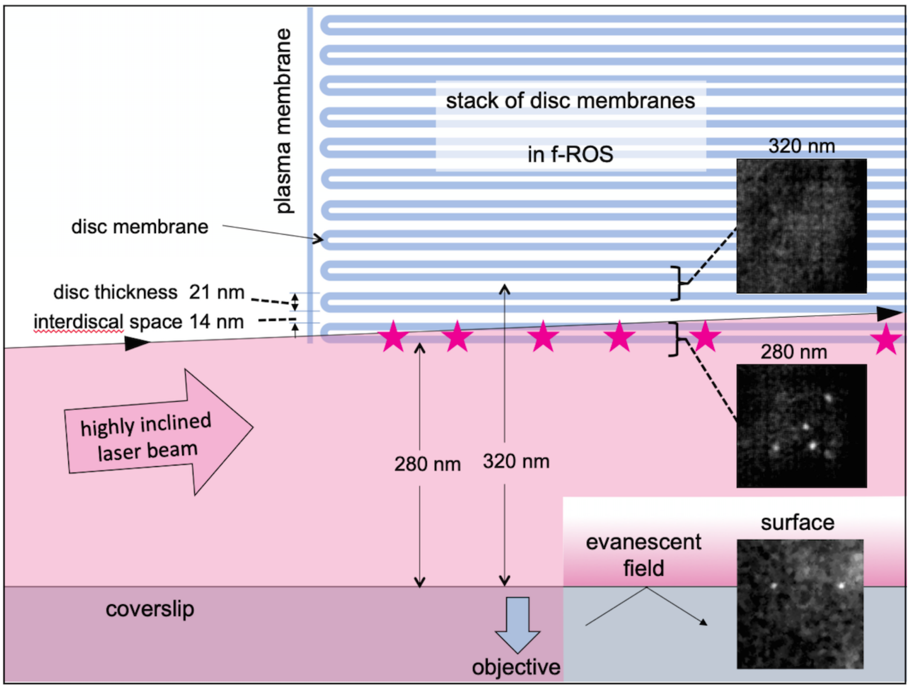


b

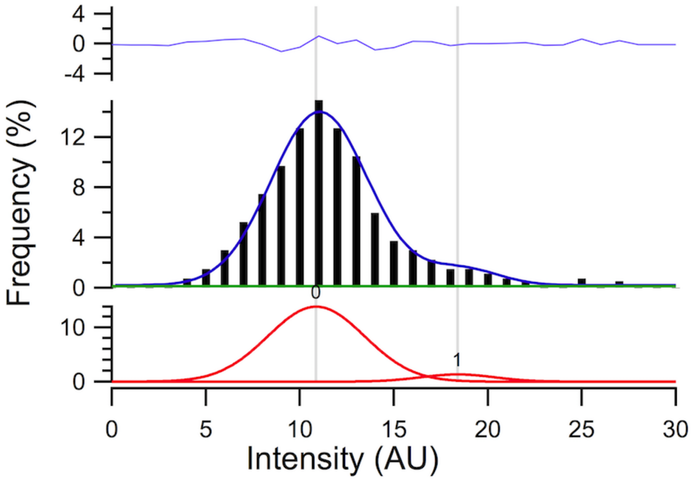


**Supplementary Figure 2 | Highly inclined epi-fluorescence microscopy enabling the observation of the molecular behaviour of rhodopsin in retinal discs. a**, Whereas the evanescent field on the glass surface permits visualization of fluorescent spots on the coverslip, fluorescent spots on the disc membrane at the

bottom of f-ROS can be observed by raising the focal plane from the glass surface by about 280 nm and by a slight increase in the incident angle. No fluorescence spots were observed when the focal plane was raised about 40 nm more, suggesting that the fluorescent spots we observed were located on one or two discs at the bottom of the f-ROS. There seems to be a transparent 280-nm gap between the glass surface and the bottom of the f-ROS, presumably ascribable to cellular exudate. **b**, The histogram showing fluorescence intensity profile of ~2,700 fluorescent spots observed in 100 trajectories in a representative disc membrane (middle; black bars). The histogram was fitted by a blue line composed of two Gaussian peaks (bottom; red lines). Top: residual. These results suggest that the bright spots originate primarily from single fluorescent molecules and that colocalisation of the two fluorescent molecules seldom occurs.

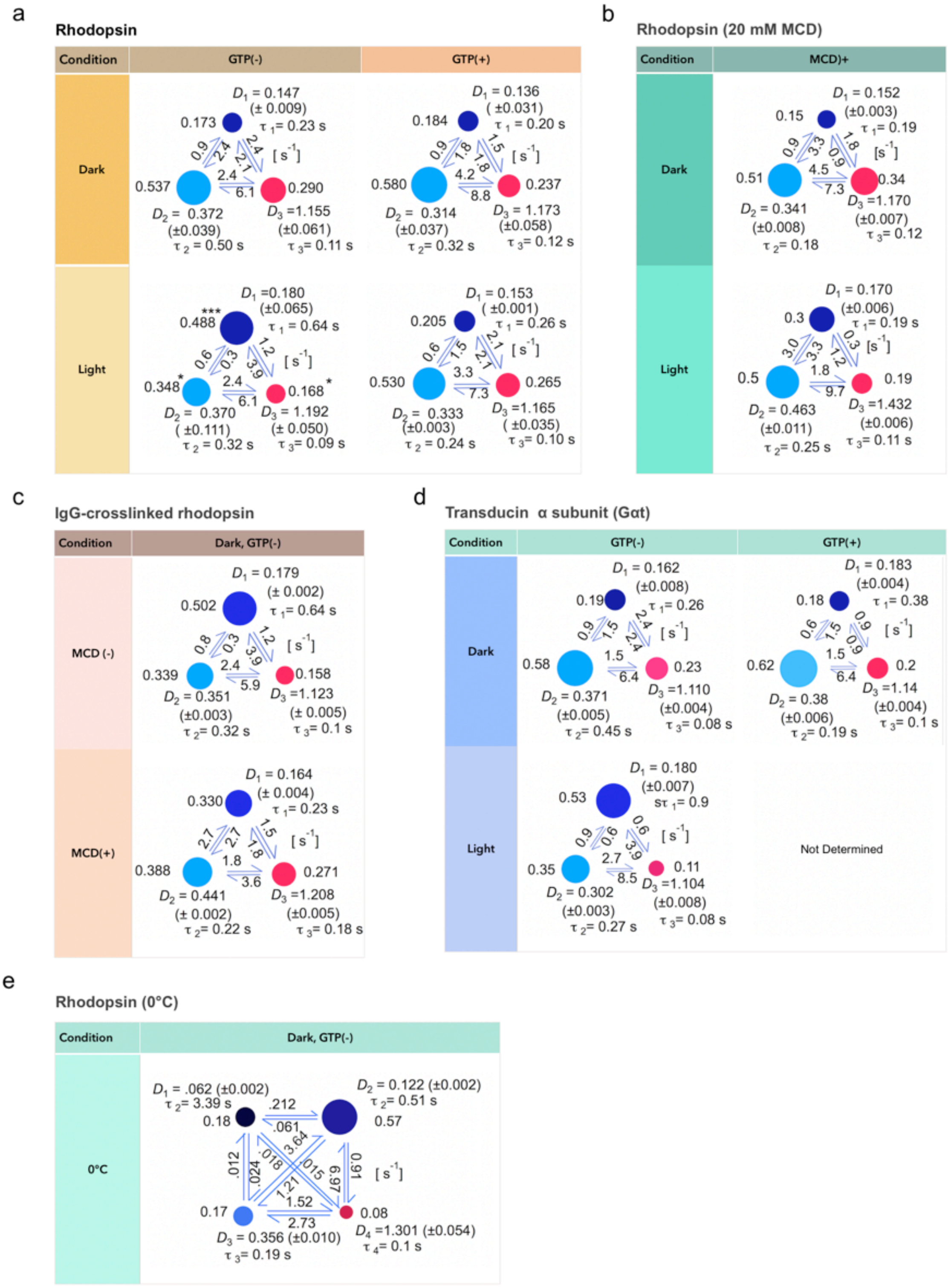


**Supplementary Figure 3 | Results summary of hidden Markov model (HMM) analysis.**

**a**, Effect of light (20% of rhodopsin isomerized) and GTP on HMM of rhodopsin. Differences are statistically significant by two-way ANOVA, *P<0.05, ***P<0.001 versus the corresponding HMM in the absence of GTP in dark-adapted disc membrane (upper-left) by Tukey's multiple-comparison test. Data are means ± SD from N=4 independent experiments (~350 trajectories; average trajectory length: ~37 data points). **b**, Effect of cholesterol-depletion by 20 mM methyl- $\beta$ -cyclodextrin on the rhodopsin HMM in the dark-adapted or light-exposed (20% rhodopsin isomerized) disc membranes. Representative HMMs are shown (~400 trajectories; average trajectory length: ~28 data points). **c**, HMM of IgG-crosslinked rhodopsin and the effect of 20 mM methyl- $\beta$ -cyclodextrin (~300 trajectories; average trajectory length: ~30 data points). **d**, Effect of light and GTP on HMM of G $\alpha_t$. Data are means ± SD from N=3 independent experiments (~300 trajectories; average trajectory length: ~30 data points). Statistically significant differences were assessed as described for rhodopsin in **a**. No optimal HMM was obtained for activated G $\alpha_t$.. **e**, Optimal HMM of rhodopsin at low temperature. A representative HMM is shown (137 trajectories; average trajectory length: ~50 data points).

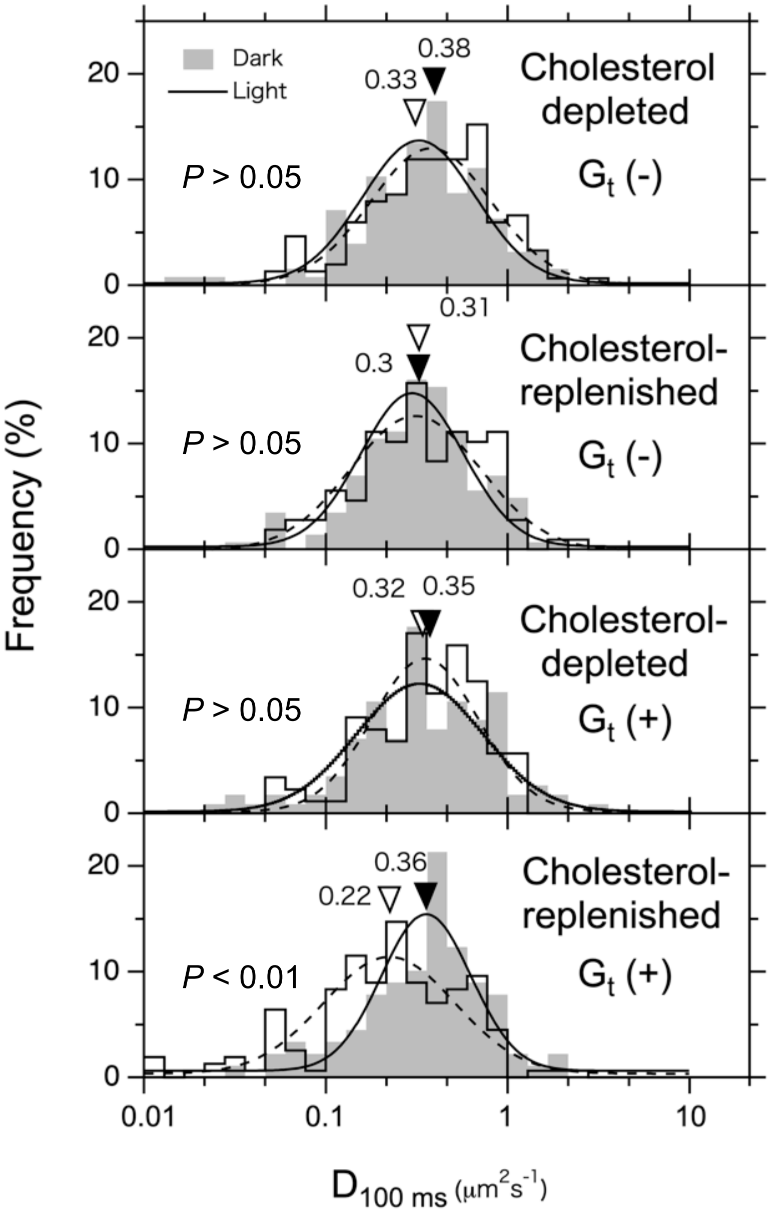


**Supplementary Figure 4 | Effects of light, cholesterol, and $G_t$ on the $D_{100ms}$ of rhodopsin.** Histograms of $D_{100ms}$ of rhodopsin in the cholesterol-depleted or -replenished disc membranes reconstituted with or without $G_t$. $G_t$ (-): $G_t$ in the dark-adapted discs were eliminated by pre-treatment of f-ROS with 20 mM MCD. $G_t$ (+): purified $G_t$ is added to 20 mM MCD-treated f-ROS. Black and white arrowheads indicate the median values of $D_{100ms}$ before and after 20% rhodopsin photo-bleaching, respectively. Solid and broken lines: curves fitted to Gaussians. Significant differences (*P*) between dark and light histograms, analysed in pairs with Mann-Whitney U test, are indicated.

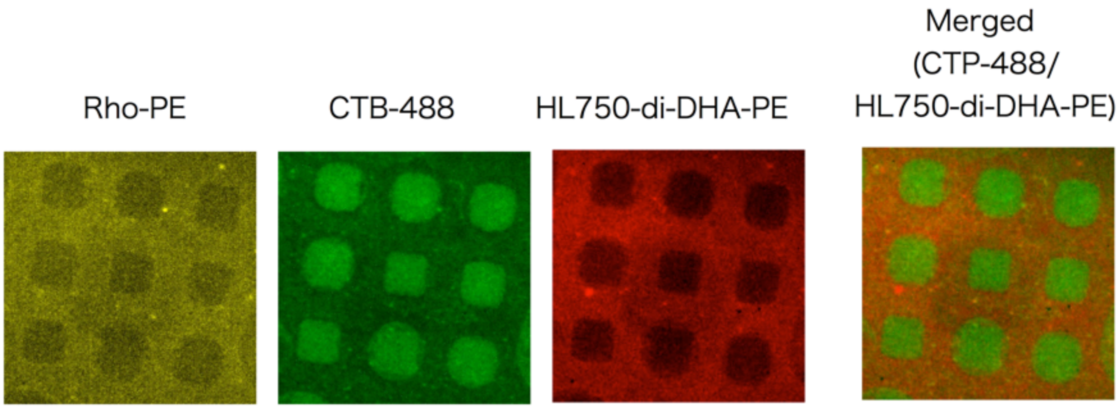


**Supplementary Figure 5 | Non-raftophilic nature of fluorescently labelled di-DHA-PE.** The non-raftophilicity of fluorescently labelled di-DHA-PE was assessed through its distribution in the non-raftophilic membrane domain, in a phase-separated lipid bilayer supported on a glass surface. Patterned separation of $L_o$ and $L_d$ phases was induced in DOPC:DPPC:Chol (1:1:1) (with GM1 and Rho-PE (1% each)) on a patterned polymeric bilayer containing polymer-free (R0) and partially polymeric (R1) regions (square-shaped pattern: $L_o$ phase, marginal area: $L_d$ phase). HL750–di-DHA-PE was introduced into the phase-separated membrane from aqueous phase. Marginal distribution of Rho-PE and HL750–di-DHA-PE, and confined distribution of cholesterol in square-shaped pattern are shown with Texas red-labelled cholera toxin B (CTB-488). Merged picture clearly indicates the non-raftophilic nature of fluorescently labelled di-DHA-PE.

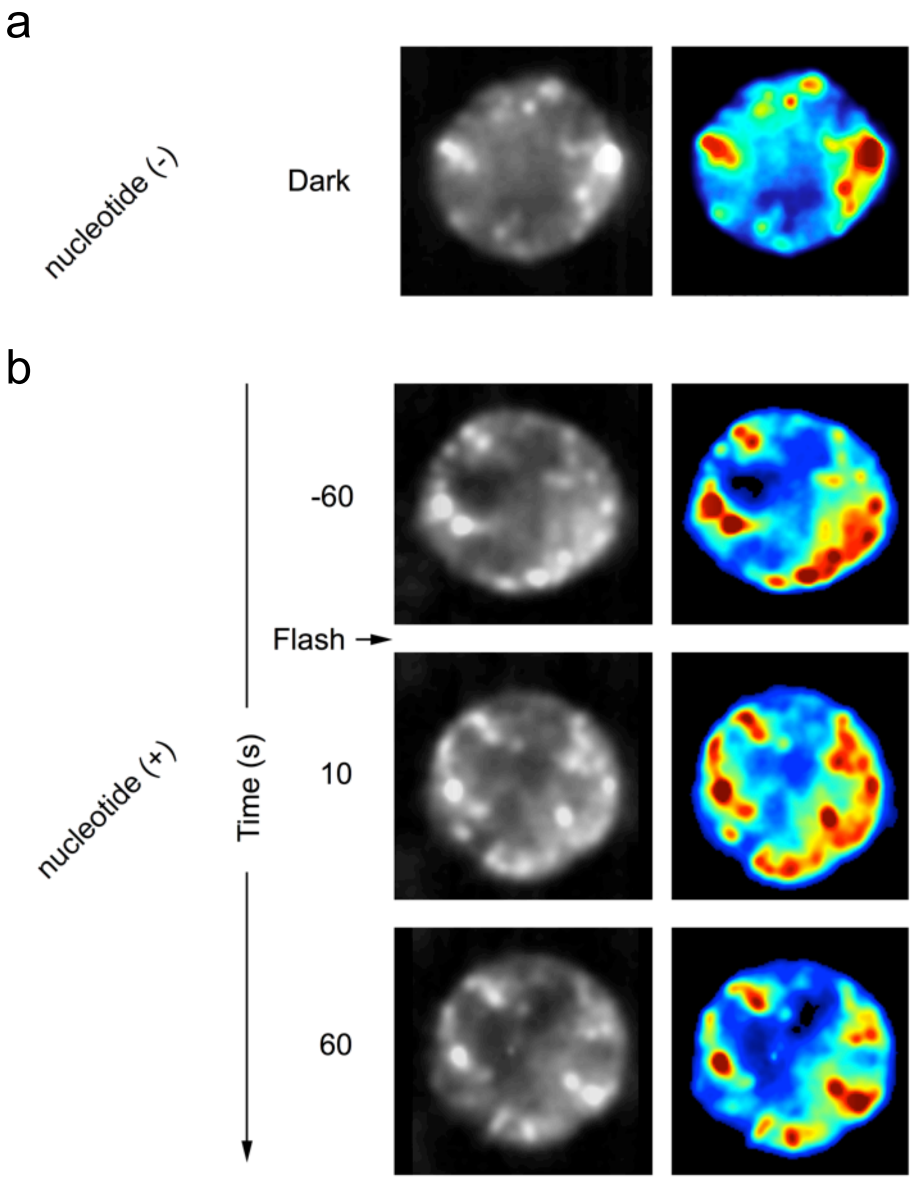


**Supplementary Figure 6 | Effect of light on PDE6 distribution. a**, An averaged image presented in grey-scale and pseudocolour obtained from a 10-s movie, indicating PDE6 distribution in a dark-adapted disc membrane in the absence of nucleotides. **b**, Averaged images presented in grey-scale and pseudocolour of 10-s movie indicating PDE6 distribution in a disc membrane before, 10 s after and 60 s after a flash isomerizing 5% of rhodopsin. 500 μM of ATP, GTP, GDP and cGMP were present. Representative data from more than three experiments are presented.

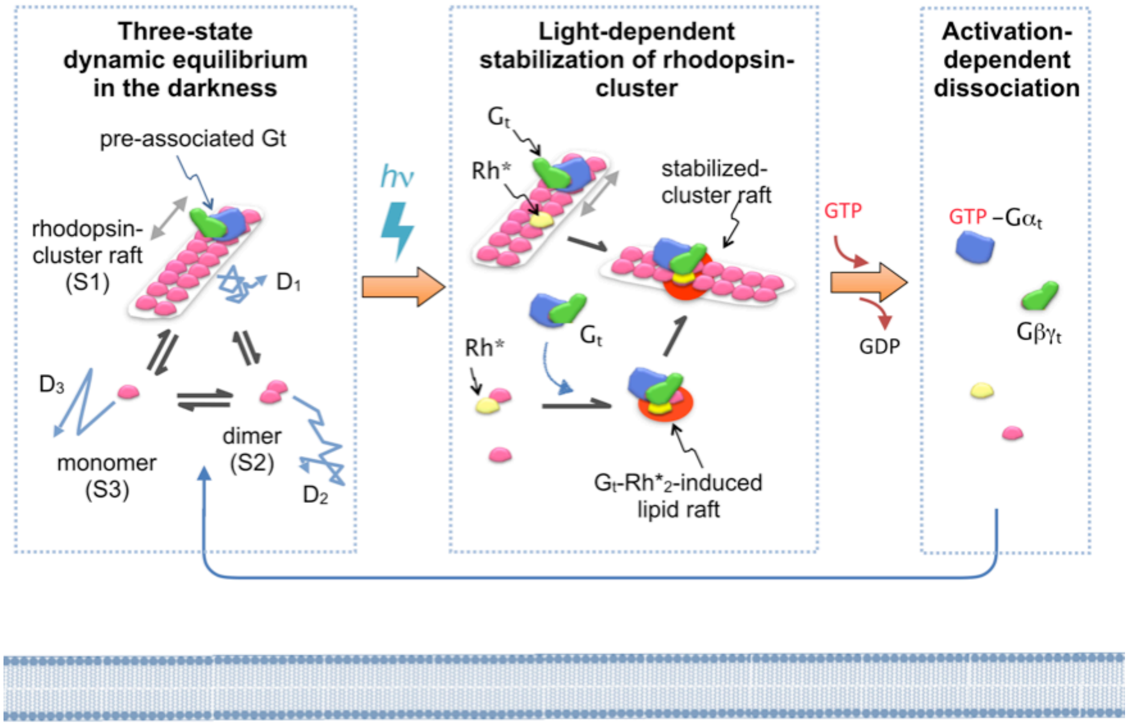


**Supplementary Figure 7 | Schematic diagram of the phototransduction system within the disc membrane.** Rhodopsin exists in dynamic equilibrium between three diffusive states S1, S2 and S3, presumably ascribable to the cluster raft, dimer and monomer of rhodopsin, having the diffusion coefficients, $D_1$, $D_2$ and $D_3$, respectively. The cluster raft was drawn as small as 1/5, or less, of the estimated size, for the sake of simplification of the figure, and the two-dimensional spread of the cluster raft was also omitted. The rhodopsin-cluster recruits raftophilic phospholipids and cholesterol, forming raftophilic membrane domain, thereby being stabilized. Cognate trimeric G protein transducin ($G_t$; $G\alpha\beta\gamma_t$) is pre-associated with rhodopsin in these three states even in darkness. If rhodopsin in the rhodopsin-cluster raft happens to be photoisomerized, pre-associated $G_t$ quickly binds to photo-excited Rh (Rh*) and the resultant $Rh^*_2$–$G_t$ stabilizes the cluster raft due to its high raftophilicity. When a rhodopsin molecule in either dimeric or monomeric form is photoisomerized, nearby $G_t$ binds to the Rh* and promotes the formation of a raftophilic Rh*2–$G_t$ complex. The complex works like a condensation nucleus, forming a rhodopsin-cluster raft. In physiological conditions, in the presence of GTP, the α-subunit of Gt ($G\alpha_t$) in the $Rh^*_2$–$G_t$ complex is activated via

nucleotide-exchange, and all components in the complex are dissociated. Dissociated rhodopsin returns to the three-state dynamic equilibrium.

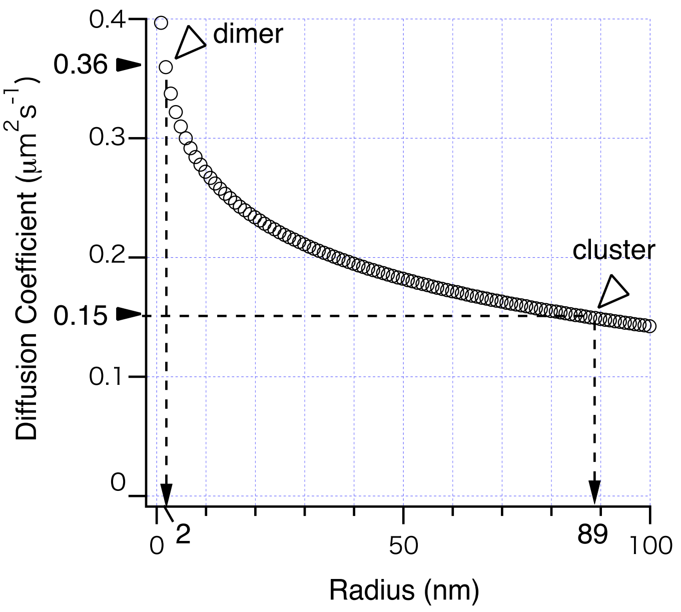


**Supplementary Figure 8 | Estimation of Rh-cluster size.** Function of the diffusion coefficient ($D_{HPW}$, y-axis) versus inclusion size (radius in nm, x-axis) calculated from Equation 1. If we assume that Rh dimers and clusters are freely diffusing in a membrane with homogeneous viscosity, Assuming that state-2 ($D_2$=3.6 $\mu m^2 s^{-1}$) corresponds to rhodopsin dimers, an extended version of the S*affman–Delbrück* equation[1] for membrane inclusions larger than 10 nm in radius, i.e.,

$$D_{\mathrm{HPW}} = \frac{k_{\mathrm{B}}T}{4\pi\mu_{\mathrm{m}}h}\left(\frac{(2\varepsilon-1)\ln(\varepsilon)-\gamma+\frac{8\varepsilon}{\pi}}{1+\frac{8\varepsilon^3\ \ln(\varepsilon)}{\pi}+\frac{a_1\varepsilon^{b1}}{1+a_2\varepsilon^{b2}}}\right) \qquad (1)$$

estimates the radius of rhodopsin-clusters in state-1 ($D_1$=1.5$\mu m^2 s^{-1}$) to be ~90 nm, where $D_{HPW}$ is the diffusion coefficient for large inclusions approximated by Hughes, Pailthorpe, and White[2], $K_B$=1.3807 × $10^{-23}$, T=293 °K, Euler's constant: $\gamma$ =0.5772, and $a_1$, $a_2$, $b_1$, and $b_2$ are constants given in a paper by Guigas and Weiss[3]; $a_1$=0.433274, $a_2$=0.670045, $b_1$=2.74819, and $b_2$=0.614465. We used a viscosity of membrane: µ=8 Poise, viscosity of aqueous phase: µ'=0.02 Poise, and membrane thickness: h=7.5 nm, as described in a previous study[4].

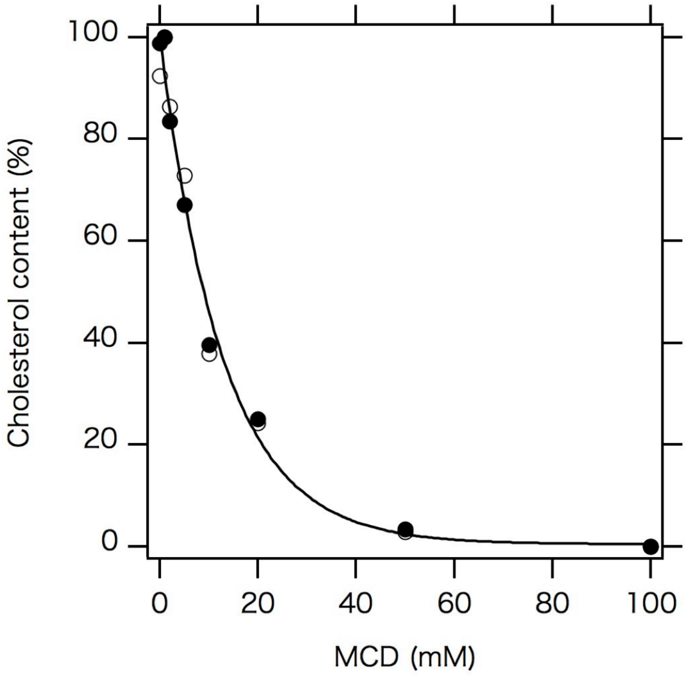


**Supplementary Figure 9 | Cholesterol content in MCD-treated disc membranes.** Cholesterol content in disc membranes (●: dark-adapted, ○: 20%-isomerized) treated with various concentration of MCD are indicated as percentages of the cholesterol content in dark-adapted discs not treated with MCD.

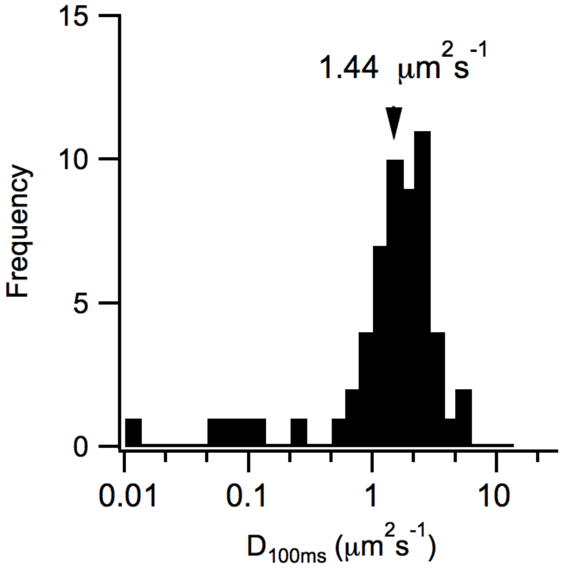


**Supplementary Figure 10 | Rapid diffusion of monomeric rhodopsin in fluid lipid bilayer membrane.** Representative histogram of the effective diffusion coefficient ($D_{100ms}$) of rhodopsin incorporated into a fluid lipid bilayer. The lipid bilayer comprised di-oleoyl-phosphatidylcholine. The arrowhead indicates the median value of $D_{100ms}$. The $D_{100ms}$ was obtained from 58 trajectories of rhodopsin.

## Supplementary Table 1

| Parameter | Posterior mean |
|---|---|
| **Num. Traj.** | 453 |
| **Ave. Traj. Length** | 27.3245 |
| **Shortest Traj.** | 15 |
| **Time Step (s)** | 0.033 |
| **Num. bootstraps.** | 100 |
| **P1** | 0.22 ± 0.02 |
| **P2** | 0.50362 ± 0.02 |
| **P3** | 0.27529 ± 0.02 |
| **D1 (μm$^2$s$^{-1}$)** | 0.1589 ± 0.0030 |
| **D2 (μm$^2$s$^{-1}$)** | 0.31013 ± 0.00392 |
| **D3 (μm$^2$s$^{-1}$)** | 1.122 ± 0019 |
| **k12 (s$^{-1}$)** | 1.948 ± 0.1432 |
| **k13 (s$^{-1}$)** | 1.748 ± 0.137 |
| **k21 (s$^{-1}$)** | 0.930 ± 0.067 |
| **k23 (s$^{-1}$)** | 4.273 ± 0.136 |
| **k31 (s$^{-1}$)** | 1.382 ± 0.111 |
| **k32 (s$^{-1}$)** | 8.758 ±0.240 |
| ***p*BS(3)*** | 0.99 |

**Supplementary Table 1 | A representative vbSPT result for rhodopsin diffusion in dark-adapted discs.** An optimal three-state model and parameters (posterior mean values± bootstrap standard error) from vbSPT analysis is presented. The dataset was obtained from an experiment with 5 dark-adapted discs in the presence of 0.5 mM GTP. *p*BS(3) denotes the bootstraps that resulted in a three-state model. * *p*BS(3)=0.99 means that 99% of the bootstraps resulted in a three-state model. The remaining 1 % was ascribed to the two-state model.

## Supplementary Table 2

| | Case 1 | | Case 2 | |
|---|---|---|---|---|
| **Parameter** | Synthetic model | Posterior mean | Synthetic model | Posterior mean |
| **Diameter (µm)** | 8 | 8 | 1 | 1 |
| **Num. Traj.** | 500 | 500 | 500 | 500 |
| **Avg. Traj. Length** | 35 | 35 | 35 | 35 |
| **Shortest Traj.** | 15 | 15 | 15 | 15 |
| **Time Step (s)** | 0.033 | 0.033 | 0.033 | 0.033 |
| **Num. bootstraps.** | - | 100 | - | 100 |
| **P1** | 0.22 | 0.246 | 0.22 | 0.22 |
| **P2** | 0.5 | 0.514 | 0.5 | 0.55 |
| **P3** | 0.28 | 0.239 | 0.28 | 0.23 |
| **D1 ($\mu m^2 s^{-1}$)** | 0.14 | 0.15 | 0.14 | 0.14 |
| **D2 ($\mu m^2 s^{-1}$)** | 0.36 | 0.35 | 0.36 | 0.32 |
| **D3 ($\mu m^2 s^{-1}$)** | 1.17 | 1.14 | 1.17 | 0.78 |
| **k12 ($s^{-1}$)** | 0.06 | 0.06 | 0.06 | 0.10 |
| **k13 ($s^{-1}$)** | 0.06 | 0.08 | 0.06 | 0.09 |
| **k21 ($s^{-1}$)** | 0.03 | 0.01 | 0.03 | 0.05 |
| **k23 ($s^{-1}$)** | 0.14 | 0.12 | 0.14 | 0.11 |
| **k31 ($s^{-1}$)** | 0.05 | 0.08 | 0.05 | 0.07 |
| **k32 ($s^{-1}$)** | 0.29 | 0.32 | 0.29 | 0.36 |
| ***p*BS** | - | 1 | - | 1 |

**Supplementary Table 2 | Validation of vbSPT with simulated data.** We validated the vbSPT for extracting HMM parameters from trajectories synthesized with realistic diffusion coefficients, reaction parameters, and an experimental length distribution obtained in single-molecule tracking of rhodopsin. Actual frog disc membrane is compartmentalized into lobules with many incisures, and the mouths of some lobules are narrow, forming a closed space of ~1 $\mu m^2$ area, though most of lobules have open mouths in the central part of the disc[5]. Therefore, we performed the validation in two cases, i.e., with a circular area of 8 µm in diameter mimicking the disc membrane (case

1) and 1 μm in diameter mimicking the closed lobule (case 2). Traj: trajectory. $P_i$: i-state occupancy. $D_i$: i-state diffusion coefficient. $k_{ij}$: rate of transition from i-state to j-state. *p*BS denotes the fraction of bootstraps yielding a three-state model as the best. In both cases, good agreement was observed between the synthetic model and the posterior mean values inferred by the vbSPT.

**References**


1. Weiß, K. *et al.* Quantifying the diffusion of membrane proteins and peptides in black lipid membranes with 2-focus fluorescence correlation spectroscopy. *Biophys. J.* **105,** 455–62 (2013).
2. Hughes, B. D., Pailthorpe, B. A. & White, L. R. The translational and rotational drag on a cylinder moving in a membrane. *J. Fluid Mech.* **110,** 349 (1981).
3. Guigas, G. & Weiss, M. Influence of hydrophobic mismatching on membrane protein diffusion. *Biophys. J.* **95,** L25-7 (2008).
4. Wang, Q. *et al.* Activation-dependent hindrance of photoreceptor G protein diffusion by lipid microdomains. *J. Biol. Chem.* **283,** 30015–30024 (2008).
5. Tsukamoto, Y. The Number , Depth and Elongation of Disc Incisures in the Retinal Rod of Rana catesbeiana. *Exp Eye Res* **45,** 105–116 (1987).
