# Supplementary material for: Raftophilic rhodopsin-clusters offer stochastic platforms for G protein signalling in retinal discs
Source: Commun Biol. 2019 Jun 14;2:209. doi: 10.1038/s42003-019-0459-6 (PMC6570657; doi:10.1038/s42003-019-0459-6)
Supplement: Supplementary file 3 — Reporting Summary [file 42003_2019_459_MOESM3_ESM.pdf]

nature research

Corresponding author(s): Fumio Hayashi

Last updated by author(s): Apr 30, 2019

Reporting Summary

Nature Research wishes to improve the reproducibility of the work that we publish. This form provides structure for consistency and transparency in reporting. For further information on Nature Research policies, see Authors & Referees and the Editorial Policy Checklist.

Statistics

For all statistical analyses, confirm that the following items are present in the figure legend, table legend, main text, or Methods section.

n/a	Confirmed	
☐	☒	The exact sample size (n) for each experimental group/condition, given as a discrete number and unit of measurement
☐	☒	A statement on whether measurements were taken from distinct samples or whether the same sample was measured repeatedly
☐	☒	The statistical test(s) used AND whether they are one- or two-sided<br>*Only common tests should be described solely by name; describe more complex techniques in the Methods section.*
☒	☐	A description of all covariates tested
☐	☒	A description of any assumptions or corrections, such as tests of normality and adjustment for multiple comparisons
☐	☒	A full description of the statistical parameters including central tendency (e.g. means) or other basic estimates (e.g. regression coefficient) AND variation (e.g. standard deviation) or associated estimates of uncertainty (e.g. confidence intervals)
☐	☒	For null hypothesis testing, the test statistic (e.g. F, t, r) with confidence intervals, effect sizes, degrees of freedom and P value noted<br>*Give P values as exact values whenever suitable.*
☐	☒	For Bayesian analysis, information on the choice of priors and Markov chain Monte Carlo settings
☐	☒	For hierarchical and complex designs, identification of the appropriate level for tests and full reporting of outcomes
☐	☒	Estimates of effect sizes (e.g. Cohen's d, Pearson's r), indicating how they were calculated

Our web collection on statistics for biologists contains articles on many of the points above.

Software and code

Policy information about availability of computer code

Data collection	No custom software was used.
Data analysis	No custom software was used.

For manuscripts utilizing custom algorithms or software that are central to the research but not yet described in published literature, software must be made available to editors/reviewers. We strongly encourage code deposition in a community repository (e.g. GitHub). See the Nature Research guidelines for submitting code & software for further information.

Data

Policy information about availability of data

All manuscripts must include a data availability statement. This statement should provide the following information, where applicable:

- Accession codes, unique identifiers, or web links for publicly available datasets
- A list of figures that have associated raw data
- A description of any restrictions on data availability

Data that support the findings of this study are available from the corresponding author upon reasonable request.

Field-specific reporting

Please select the one below that is the best fit for your research. If you are not sure, read the appropriate sections before making your selection.

☒ Life sciences ☐ Behavioural & social sciences ☐ Ecological, evolutionary & environmental sciences

For a reference copy of the document with all sections, see nature.com/documents/nr-reporting-summary-flat.pdf

Life sciences study design

All studies must disclose on these points even when the disclosure is negative.

Sample size	Sample size for variational hidden Markov model analysis was initially determined on the basis of that used in the original paper on vbSPT (http://www.ncbi.nlm.nih.gov/pubmed/23396281). And then, we validated the data size by repeated vbSPT analysis.
Data exclusions	In the mean-square-displacement analysis (MSD analysis) we excluded the tracking data which gave no good enough linear fitting in MSD-time interval plot at the range of time interval within 300 ms, because this deviation could be due to the confinement of single-molecule in small lobules characteristic to frog retinal discs. We described this in Method Section.
Replication	All attempts at replication were successful.
Randomization	Our experiments were done in vitro.
Blinding	Blinding was not possible. Our experiments were done in vitro.

Reporting for specific materials, systems and methods

We require information from authors about some types of materials, experimental systems and methods used in many studies. Here, indicate whether each material, system or method listed is relevant to your study. If you are not sure if a list item applies to your research, read the appropriate section before selecting a response.

Materials & experimental systems

n/a	Involved in the study	
☐	☒	Antibodies
☒	☐	Eukaryotic cell lines
☒	☐	Palaeontology
☐	☒	Animals and other organisms
☒	☐	Human research participants
☒	☐	Clinical data

Methods

n/a	Involved in the study	
☒	☐	ChIP-seq
☒	☐	Flow cytometry
☒	☐	MRI-based neuroimaging

Antibodies

Antibodies used	A highly specific mAb 1D4 for rhodopsin was purchased from Univ British Colombia. Anti-PDE6 polyclonal antibody was raised against a peptide corresponding to 137Asp-156Val of Rana pipience.
Validation	Specificity and affinity of antibody 1D4 has been established. Anti-PDE6 antibody can stain a single band corresponding to PDE6 in Western Blotting.

Animals and other organisms

Policy information about studies involving animals; ARRIVE guidelines recommended for reporting animal research

Laboratory animals	*For laboratory animals, report species, strain, sex and age OR state that the study did not involve laboratory animals.*
Wild animals	We used wild bull frogs (Rana catesbeiana) for experiments.
Field-collected samples	*For laboratory work with field-collected samples, describe all relevant parameters such as housing, maintenance, temperature, photoperiod and end-of-experiment protocol OR state that the study did not involve samples collected from the field.*
Ethics oversight	This study was was approved by the Institutional Animal Care and Use Committee, and carried out according to Kobe University's animal experimentation regulations.

Note that full information on the approval of the study protocol must also be provided in the manuscript.
