# Supplementary material for: Raftophilic rhodopsin-clusters offer stochastic platforms for G protein signalling in retinal discs
Source: Commun Biol. 2019 Jun 14;2:209. doi: 10.1038/s42003-019-0459-6 (PMC6570657; doi:10.1038/s42003-019-0459-6)
Supplement: Supplementary file 4 — Description of additional supplementary items [file 42003_2019_459_MOESM4_ESM.doc]

**Supplementary Movies**

**Supplementary Movie 1. Single-molecule tracking of rhodopsin.** A representative movie of single fluorescent molecules of HL750–Fab'–1D4 bound to the dark-adapted disc membrane in the presence of 500 µM GTP. Frame rate: 30 s^-1^. Trajectories are color-coded with trajectory indices. Scale bar: 2 µm.

**Supplementary Movie 2. Dynamic clustering of rhodopsin in a disc membrane.** Dark-adapted disc membrane was incubated with 26 nM HL750–Fab'–1D4. Frame rate: 30 frames s^-1^. Scale Bar:2 µm. GTP was absent.

**Supplementary Movie 3. Annular distribution of di-DHA-PE in a disc membrane.** Dark-adapted disc membrane was incubated with 4.8 nM HL750–di-DHA-PE. Frame rate: 30 frames s^-1^. Scale bar: 2 µm. Nucleotides were absent.

**Supplementary Movie 4. Single-molecule trajectories of di-DHA-PE in a disc membrane.** Dark-adapted disc membrane was incubated with 0.2 nM HL750–di-DHA-PE. Frame rate: 30 frames s^-1^. Scale bar: 2 µm. Nucleotides were absent.

**Supplementary Movie 5. Dynamic clustering of G_t_ in a disc membrane.** Dark-adapted disc membrane was incubated with 30 nM HL750–Gα_t_ and Gβγ_t_. Frame rate: 30 frames s^-1^. Scale bar: 2 µm. A bright spot on the upper-left is likely the axoneme. GTP was absent.

**Supplementary Movie 6. Behaviour of PDE6 in a disc membrane.** Dark-adapted frog disc membrane was incubated with 30 nM HL750–Fab' specific to the GAF1 domain of PDE6 α-subunit. No nucleotide was present. Scale bar: 2 µm. Frame rate: 30 frames s^-1^.

**Supplementary Data 1.** Raw data used to generate the graphs presented in the manuscript.
